# MALAT1: An Epigenetic Regulator of Inflammation in Diabetic Retinopathy

**DOI:** 10.1038/s41598-018-24907-w

**Published:** 2018-04-25

**Authors:** Saumik Biswas, Anu Alice Thomas, Shali Chen, Erfan Aref-Eshghi, Biao Feng, John Gonder, Bekim Sadikovic, Subrata Chakrabarti

**Affiliations:** 1 Department of Pathology and Laboratory Medicine, Western University, London, Ontario Canada; 2 Department of Ophthalmology, Western University, London, Ontario Canada

## Abstract

Despite possessing limited protein-coding potential, long non-coding RNAs (lncRNAs) have been implicated in a myriad of pathologic conditions. Most well documented in cancer, one prominent intergenic lncRNA known as MALAT1 is notorious for its role in impacting epigenetic mechanisms. In this study, we established a novel epigenetic paradigm for MALAT in diabetic retinopathy (DR) by employing siRNA-mediated MALAT1 knockdown in human retinal endothelial cells (HRECs), a *Malat1* knockout animal model, vitreous humor from diabetic patients, pharmacological inhibitors for histone and DNA methylation, RNA immunoprecipitation, western blotting, and a unique DNA methylation array to determine glucose-related alterations in *MALAT1*. Our findings indicated that MALAT1 is capable of impacting the expressions of inflammatory transcripts through its association with components of the PRC2 complex in diabetes. Furthermore, the vitreous humors from diabetic patients revealed increased expressions of MALAT1, TNF-α, and IL-6. Intriguingly, our DNA methylation array demonstrated that transient high glucose exposure in HRECs does not contribute to significant methylation alterations at CpG sites across the *MALAT1* gene. However, global inhibition of DNA methyltransferases induced significant increases in MALAT1 and associated inflammatory transcripts in HRECs. Our findings collectively demonstrate the importance of MALAT1 in inflammation and epigenetic regulation in DR.

## Introduction

As the global prevalence of diabetes is projected to rise to 642 million by 2040, there is an urgent need for understanding the pathogenesis of diabetic complications to develop effective therapeutic agents^1,2^. Diabetic retinopathy (DR), a debilitating ocular complication, is the leading cause of blindness among working-aged adults in industrialized nations^3^. The asymptomatic nature of DR, prior to the development of vision loss, is concerning, as nearly all type 1 diabetic patients and over 60% of type 2 diabetic patients will develop evidence of retinopathy within the first 20 years of diagnosis^4,5^. Despite the presence of management strategies, the rate of DR is still expected to rise due to the increasing incidence of diabetes, which necessitates the need for exploration of new molecular aspects of DR to expand the current scope of therapy.

In the last two decades, the rapid advent of high-throughput genomic technology has made it evident that more than 97% of the human genome is comprised of non-protein-coding elements, such as non-coding RNAs (ncRNAs)^6^. Although significant research has been conducted in annotating the transcripts that arise from these genomic regions, a vast amount of information regarding the roles and functions of ncRNAs in DR remains elusive.

Long non-coding RNAs (lncRNAs) are a class of ncRNAs that are greater than 200 nucleotides in length and have diverse roles in a myriad of cellular processes including the ability to repress the expression of nearby protein-coding genes^7^, X-chromosome inactivation^8^, and the modulation of protein activity^9^. In DR, transcriptomic analyses have identified more than 300 lncRNAs that display aberrant expression profiles in the retina—with over 80 lncRNAs being overexpressed^10^. Among these upregulated lncRNAs, MALAT1 (metastasis-associated lung adenocarcinoma transcript 1) is a prominent intergenic lncRNA that is known to be associated with metastasis in non-small cell lung cancer (NSCLC)^11^. Since it is accepted that endothelial cells (ECs) are main targets of diabetes-induced tissue damage, recent research has also revealed novel roles for MALAT1 in diabetic complications. Results from our previous study indicate that MALAT1 knockdown in human umbilical vein endothelial cells (HUVECs), under hyperglycemic conditions, down-regulates serum amyloid antigen 3 (SAA3) activation, subsequently reducing the RNA and protein expressions of key inflammatory mediators (IL-6 and TNF-α) implicated in diabetic complications^12^. Further, augmentation of MALAT1 expression by hypoxia promotes a proliferative response in HUVECs^13^.

In order to understand how lncRNAs, such as MALAT1, regulate the inflammatory processes underpinning these pathologies, the complex molecular interplay between lncRNAs and other epigenetic events must be examined in an integrated way. Several cancer-related studies have revealed that MALAT1 is capable of binding to enhancer of zeste homolog 2 (EZH2), the main catalytic subunit of the histone methyltransferase polycomb repressive complex 2 (PRC2), and promotes oncogenesis by reprograming the chromatin state^14–17^. Furthermore, in the context of DNA methylation, it has been previously reported that lung cancer tissues exhibit reduced methylation in the *MALAT1* promoter, which subsequently enhances MALAT1 expression^18^. However, in contrast, others have reported minimal methylation alterations at the CpG island in the *MALAT1* promoter of esophageal squamous cell carcinoma tissues and concluded that CpG island methylation status may not contribute to MALAT1 dysregulation^19^. Nevertheless, despite the recent emergence of these epigenetic roles for MALAT1 in cancer, the question of whether MALAT1 influences other epigenetic mediator proteins to regulate inflammation in DR still remains unanswered.

Here, we first determined the expression level of MALAT1 in human retinal microvascular endothelial cells (HRECs) cultured in high glucose (HG) and subsequently analyzed the expressions of common inflammatory markers (IL-6, TNF-α, MCP-1, and IL-1β) along with components of PRC2 (EZH2, SUZ12, and EED) to represent histone methyltransferase activity. Following our initial findings, we employed MALAT1 knockdown and *Malat1* knockout (KO) strategies in HRECs and in a mouse model, respectively, to determine the functional role and significance of MALAT1 on inflammation and PRC2 activity in DR. Moreover, to substantiate the data from our *in vitro* and *in vivo* animal experiments, we examined MALAT1 and its associated inflammatory markers in the vitreous humor (VH) of diabetic patients undergoing vitrectomy. We also examined MALAT1 binding to EZH2 by RNA immunoprecipitation in HRECs. As well, we further investigated the effects of HG on CpG island methylation status in the *MALAT1* promoter of HRECs using a methylation array and then explored the impact of specific treatment(s) targeting MALAT1, histone methyltransferases, and DNA methyltransferases (DNMTs).

## Results

### MALAT1 is upregulated in HRECs exposed to high glucose

Retinal ECs are a fundamental cell type in the retinal microvasculature^20^. Retinal ECs are also one of the earliest cells to undergo glucose-induced damage as a consequence of DR^21^. Hence, we used HRECs for our *in vitro* experiments. Of note, we have previously reported HG-induced increases in the expression of MALAT1 in large vessel ECs^12^. In order to investigate the differential expression patterns of MALAT1 and inflammatory markers, we cultured HRECs in HG and examined MALAT1, IL-6, TNF-α, MCP-1 and IL-1β RNA expressions at 12, 24, 48, and 72 hours. RT-qPCR analyses demonstrated that expressions of MALAT1 and inflammatory markers in HRECs peaked at 48 hours following HG incubation (see Fig. S1A–E). Furthermore, no significant differences for MALAT1, IL-6, TNF-α, MCP-1 and IL-1β expressions were observed osmotic controls (data not shown). Following our findings, we decided to use the 48-hour time point for our subsequent *in vitro* experiments.

### MALAT1 knockdown prevents augmented production of inflammatory cytokines and PRC2 components *in vitro*

**Figure 1:**
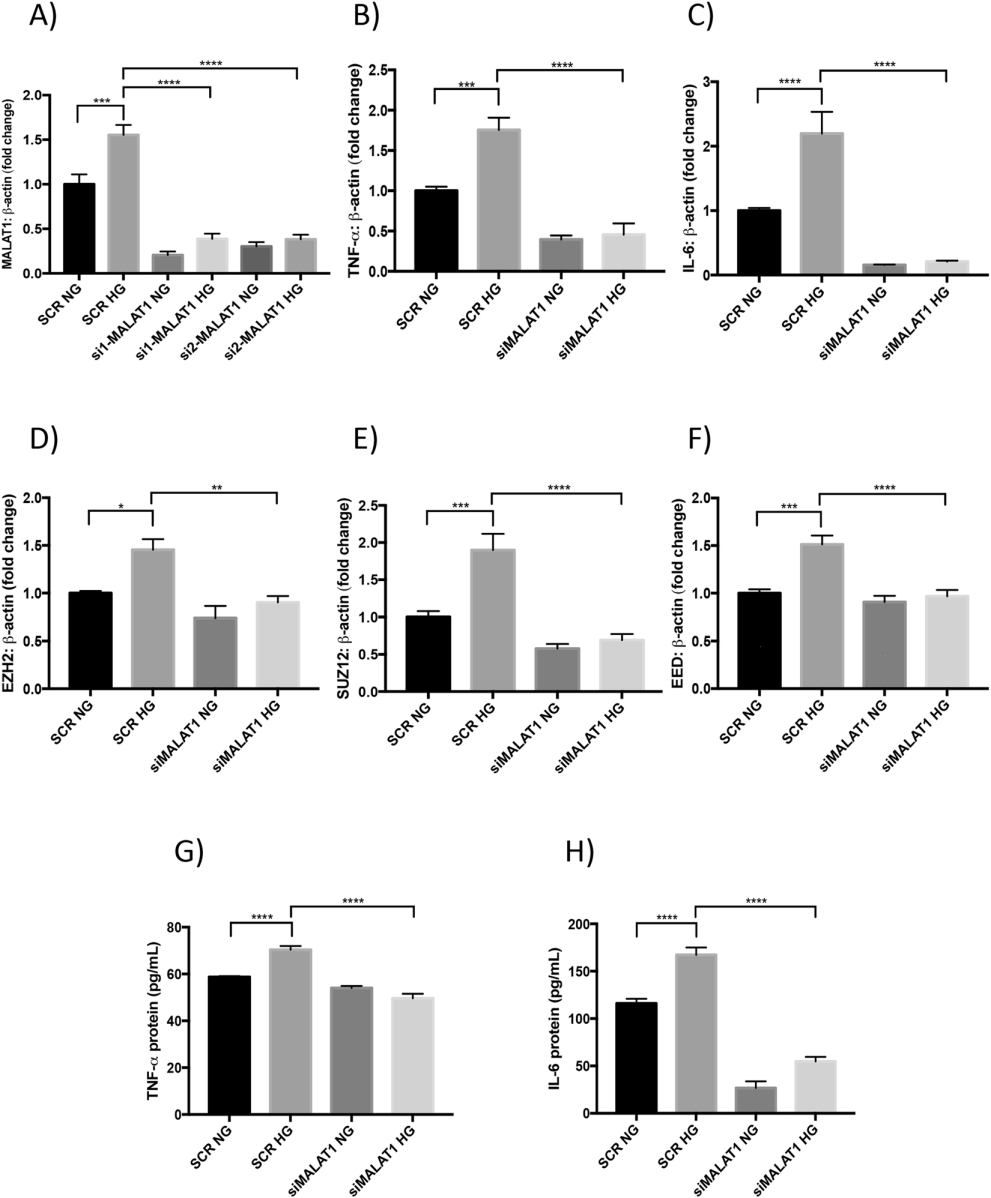
MALAT1 regulates glucose-induced production of inflammatory cytokines and PRC2 components *in vitro*. RT-qPCR analyses indicating HG-induced upregulation of (**A**) MALAT1 transcript, (**B**,**C**) proinflammatory transcripts (TNF-α, IL-6), and (**D**–**F**) PRC2 components (EZH2, SUZ12, and EED) in HRECs. SiMALAT1 transfections caused significant reductions in glucose-induced upregulation of these transcripts. (**G**,**H**) ELISAs for protein levels of IL-6 and TNF-α (expressed as pg/mL) demonstrated prevention of glucose-induced increases of these peptides following siRNA transfection (data expressed as a ratio to β*-actin* (mean ± SEM); normalized to SCR NG; *P < 0.05, **P < 0.01, ***P < 0.001, and ****P < 0.0001, compared to SCR NG or SCR HG; *n* = 6 from three independent experiments and performed in triplicates; SCR = scrambled siRNA; NG = 5 mM D-glucose; HG = 25 mM D-glucose; si1-MALAT1 (MALAT1 siRNA 1, ID: n272231); and si2-MALAT1 (MALAT1 siRNA 2, ID: n272233)).

After observing upregulation of MALAT1 at 48 hours *in vitro*, we wanted to delineate the functional importance of the MALAT1 transcript in inflammation and the role of PRC2 complex expression in HRECs. We, therefore, employed a siRNA-mediated knockdown approach targeting MALAT1. RT-qPCR analyses confirmed a ~75% reduction in total MALAT1 RNA after siRNA treatments when compared to scrambled controls (Fig. 1A). MALAT1 silencing in HG-treated HRECs dramatically reduced the overall expression of tumor necrosis factor-alpha (TNF-α) and interleukin-6 (IL-6) by a ~74% and a ~93% reduction, respectively, when compared to scrambled controls (Fig. 1B,C). Similarly, IL-1β and MCP-1 RNA expressions were also significantly reduced following siMALAT1 treatment (Fig. S2A,B). We further expanded our investigation to include the effects on inflammatory proteins after siMALAT1 treatment and findings from the ELISAs revealed that after knocking down MALAT1 in a HG environment, IL-6 and TNF-α protein levels significantly decreased when compared to scrambled HG controls (Fig. 1G,H). The trends observed from these experiments are consistent with our previous findings of decreased expressions for IL-6 and TNF-α after siMALAT1 transfection in HUVECs^12^.

To examine whether MALAT1 knockdown can affect the components of PRC2, we analyzed EZH2, SUZ12, and EED RNA levels in HRECs following siMALAT1 transfection. As compared with scrambled controls, we found significantly reduced EZH2, EED, and SUZ12 RNA levels in HG-treated HRECs transfected with MALAT1 siRNA (Fig. 1D–F). Moreover, to demonstrate MALAT1’s ability to impact PRC2 at the protein level, we performed western blotting and examined EZH2 expression after MALAT1 knockdown. Our western blot analyses confirmed that EZH2 protein expressions were reduced in HRECs transfected with siMALAT1 (Fig. S2C). In addition to our western blot data, we performed RNA immunoprecipitation and confirmed MALAT1 binding with EZH2. In fact, MALAT1 RNA was significantly enriched in the EZH2-antibody precipitated RNA fraction from HG-treated HRECs compared to controls (Fig. S2D).

Furthermore, extending our knockdown analyses, we observed significant positive correlations between MALAT1 RNA expression and the RNA expressions of PRC2 components in HRECs subjected to HG and siMALAT1 treatments (two-sided Pearson correlation; Fig. S3A–C). As well, positive correlations existed between EZH2 RNA expression and the RNA expressions of inflammatory markers, IL-6 and TNF-α, in the siMALAT1 + HG group (Fig. S3D,E). Collectively, our findings from the knockdown experiment indicate that MALAT1 RNA levels play an important role in influencing glucose-induced upregulation of inflammatory cytokines and components of PRC2 in HRECs.

### *Malat1* knockout alleviates diabetes-induced retinal inflammatory cytokines and elevated PRC2 expression

With the genetic ablation of the *Malat1* gene not contributing to noticeable developmental effects in mice under basal homeostatic conditions^22–24^, we decided to use a diabetic *Malat1* KO mice model in order to evaluate the direct function of the *Malat1* gene on inflammation and PRC2 expression in retinal tissues. *Malat1* KO mice with STZ-induced diabetes and age-and sex-matched controls were monitored for 2 months. WT-diabetic (WT-D) mice showed hyperglycemia and reduced body weight gain (Table S2). Polyuria and glucosuria were also observed in diabetic mice (data not shown). Nevertheless, no effects on these parameters were seen following *Malat1* nullification.

**Figure 2:**
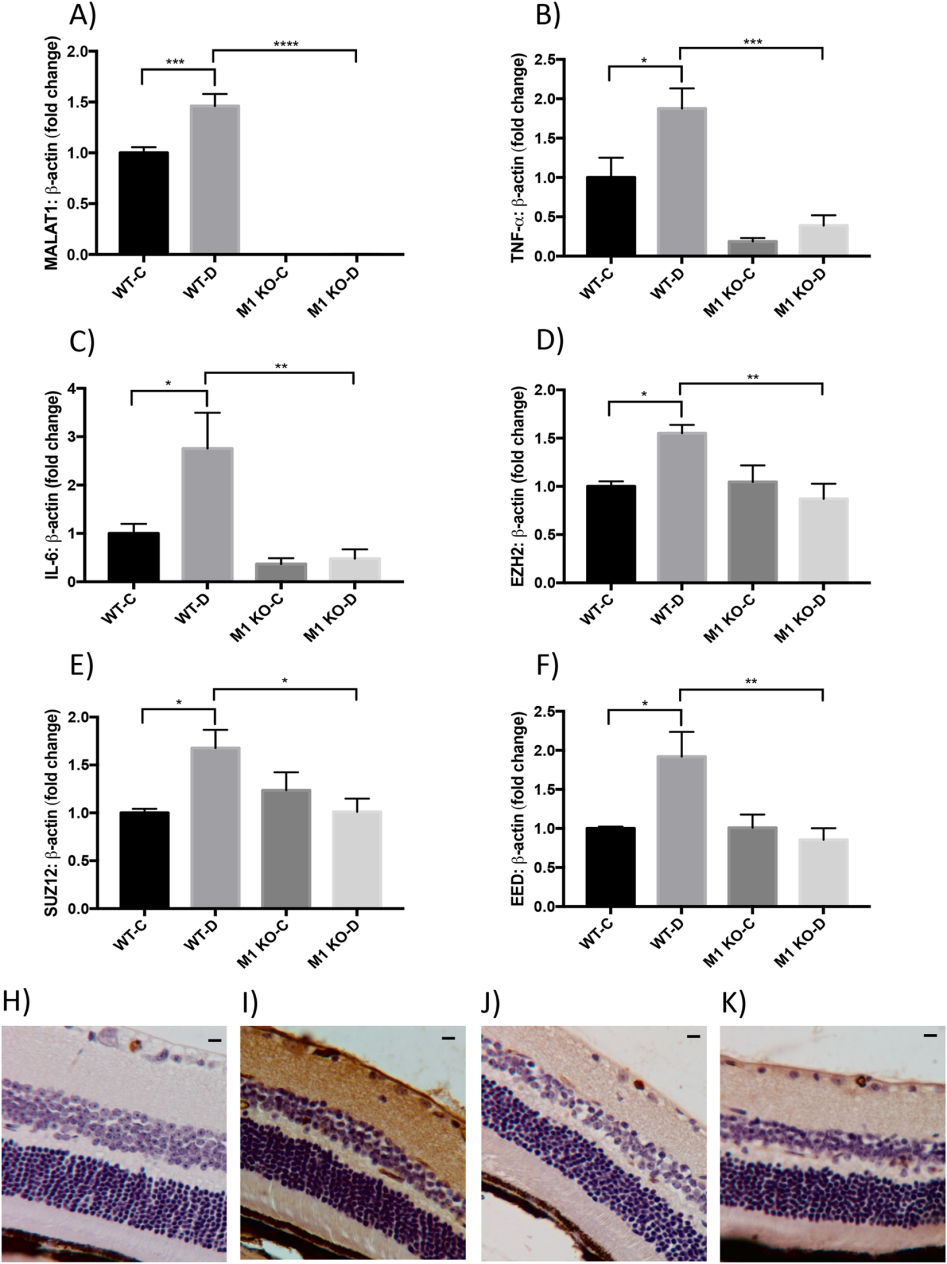
*Malat1* knockout alleviates diabetes-induced retinal inflammatory cytokines, elevated PRC2 expression, and IgG leakage *in vivo*. RT-qPCR analyses of the retinas from animals, following two months of poorly controlled diabetes showed increased expressions of (**A**) MALAT1, (**B**,**C**) inflammatory transcripts (TNF-α, IL-6), and (**D**,**E**,**F**) PRC2 components (EZH2, SUZ12, and EED) in WT-D retinas compared to WT-C retinas. *Malat1* KO prevented such increases in the M1 KO-D group (data expressed as a ratio to β*-actin* (mean ± SEM); normalized to WT-C; *P < 0.05, **P < 0.01, ***P < 0.001, and ****P < 0.0001, compared to WT-C or WT-D; *n* = 6/group). (**H**–**K**) IgG staining shows elevated IgG leakage in I) WT-D retinas (score 3) and reduced leakage in the (K) M1 KO-D retinas (score 1). No changes in IgG leakage were observed between (H) WT-C (score 0) and (J) M1 KO-C (score = 1) animals (scale bar = 10 μM). WT-C = Wild-type control; WT-D = Wild-type diabetic; M1 KO-C = *Malat1* KO control; and M1 KO-D = *Malat1* KO diabetic.

Our analyses demonstrated that MALAT1 RNA expression was significantly elevated (more than ~0.46-fold) in WT-D mice retinas (Fig. 2A) compared to WT non-diabetic control mice (WT-C). MALAT1 RNA expressions were nearly non-existent in both *Malat1* KO animal groups (M1 KO-C and M1 KO-D), confirming that MALAT1 transcripts are depleted in the retinal tissues of this global knockout model (Fig. 2A). Moreover, our initial findings of MALAT1 upregulation in WT-D mice retinas are consistent with previous trends of increased MALAT1 expression in retinas of diabetic rats^25,26^.

Intriguingly, upon *Malat1* gene inactivation, significant RNA expression changes were observed among IL-6, TNF-α, IL-1β, MCP-1, EZH2, EED, and SUZ12 in retinal tissues of diabetic mice (Figs 2B–F, S4A,B). When compared to WT-D animals, all inflammatory transcripts were significantly downregulated (>50%) in *Malat1* KO diabetic animals (Figs 2B,C, S4A,B). These changes were associated with downregulation of transcripts of PRC2 components in *Malat1* KO diabetic animals (Fig. 2D–F). Together, our data suggests that presence of the *Malat1* gene is important in regulating diabetes-induced inflammation and PRC2 components in retinal tissues

### *Malat1* knockout diminishes vascular leakage in the diabetic retina

Damage to the blood-retinal barrier (BRB) becomes imminent during the severe stages of DR, which can consequently lead to irreparable vision damage^27^. In fact, risk for developing vision-threatening complications heightens when a chronic hyperglycemic environment allows for increased extravasation of large plasma proteins into the neural retina^27,28^. Therefore, to examine functional alterations in our diabetic animal model, we used IgG staining on retinal tissues (Fig. 2H–K). Both in WT-C and M1 KO mice, IgG was mostly limited within the capillaries without any significant staining of retinal tissues (scores 0–1; Fig. 2H,J). In contrast, diffuse staining of retinal tissues (score 3) were seen in WT-D animals (Fig. 2I). Such changes were prevented in M1 KO-D mice (score 1; Fig. 2K). Taken together, these findings support the theory that MALAT1 is implicated in advancing BRB breakdown.

### MALAT1 is upregulated and associated with increased inflammatory markers in the vitreous of diabetic patients

**Figure 3:**
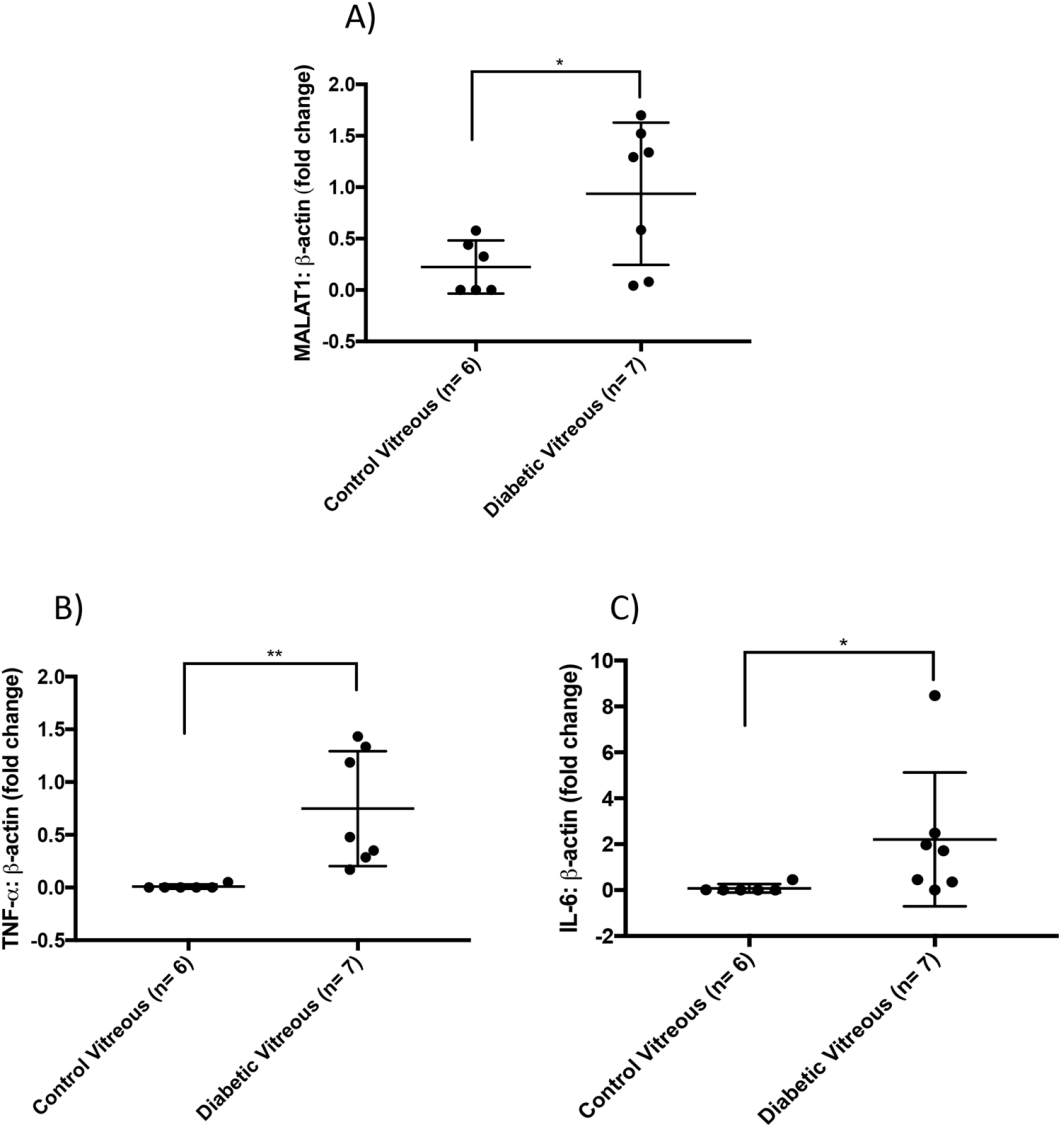
MALAT1 upregulation is associated with increased inflammatory markers in the vitreous of diabetic patients. RT-qPCR analyses of the vitreous humors indicate that diabetic patients have elevated expressions of (**A**) MALAT1, (**B**) TNF-α, and (**C**) IL-6 transcripts (data expressed as a ratio to β*-actin* (mean ± SD); *P < 0.05 and **P < 0.01, compared to control vitreous).

Alteration in the VH composition ultimately reflects the retinal environment^29^. In individuals with late stage DR, there are increased concentrations of proinflammatory cytokines and soluble growth factors in the VH that mediate retinal neovascularization within the eye^30–32^. Therefore, we decided to examine RNA levels of MALAT1 and its potential downstream molecules, IL-6 and TNF-α in the diabetic VH. RT-qPCR analyses revealed that MALAT1 expression was significantly upregulated in the vitreous of PDR patients than that of non-diabetic patients (Fig. 3A). PDR patients also demonstrated significant upregulations of TNF-α and IL-6 in the VH when compared to the vitreous of non-diabetic patients without retinopathy (Fig. 3B,C). In summary, our findings suggest that MALAT1 upregulation in the diabetic vitreous is associated with a pathogenetic state.

### Histone methylation impacts MALAT1 and some of its downstream targets

**Figure 4:**
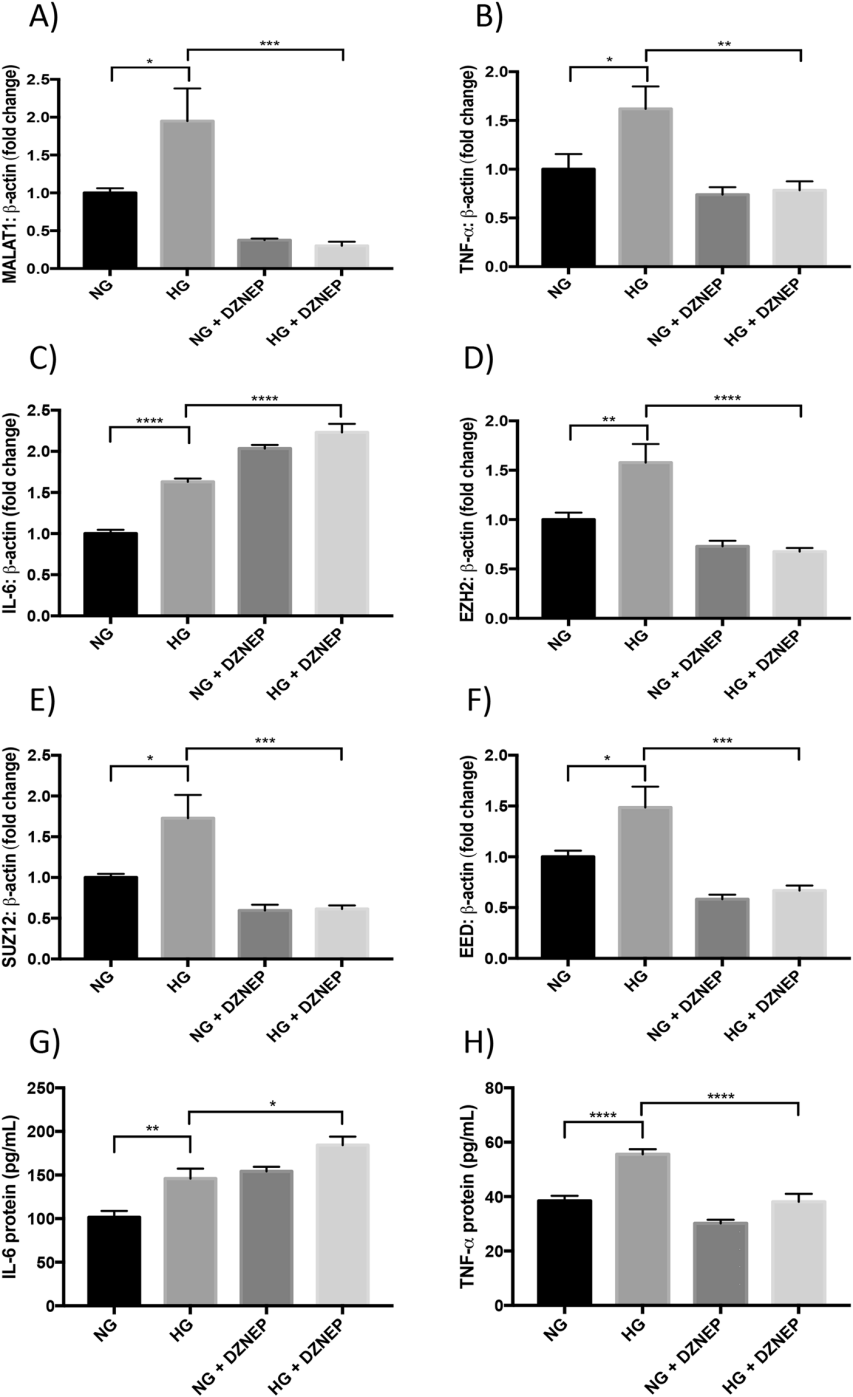
Global methylation inhibitor (DZNep) prevents glucose-induced upregulation of MALAT1, TNF-α, and PRC2 expressions. RT-qPCR findings indicate HG-induced elevations of (**A**) MALAT1, (**B**,**C**) inflammatory transcripts (TNF-α, IL-6), and (**D**–**F**) PRC2 components (EZH2, SUZ12, and EED) compared to NG at 48 hours. Such upregulations were prevented (except IL-6) following DZNep treatment. Protein levels of (**G**) IL-6 and (**H**) TNF-α showed similar patterns (RNA data expressed as a ratio to β*-actin* (mean ± SEM); normalized to NG; *P < 0.05, **P < 0.01, ***P < 0.001, and ****P < 0.0001, compared to NG or HG; *n* = 6 from three independent experiments and performed in triplicates; and protein data are expressed as pg/mL).

With epigenetic reprogramming by pharmacological intervention receiving critical recognition and showing considerable promise in cancer trials^33,34^, we decided to explore the function of DZNep on HRECs and elucidate the effects of inhibiting histone methyltransferases on MALAT1 and inflammation in a diabetic environment. Following HG incubation, DZNep-treated HRECs demonstrated significant reductions in the RNA expressions of EZH2, EED, and SUZ12, compared to HG-treated HRECs (Fig. 4D–F). Accompanying the reduction of PRC2 components, HRECs in HG following DZNep treatment showed significant reductions in MALAT1 and TNF-α RNA expressions when compared to control HG-treated HRECs (Fig. 4A,B). However, this is in stark contrast to the trends observed for IL-6, IL-1β, and MCP-1; in which, the RNA expressions of these transcripts were significantly upregulated after DZNep treatment in both NG-and HG-treated HRECs, as compared to control HG-treated HRECs (Figs 4C, S4C,D).

In order to further verify this surprising observation of IL-6, IL-1β, and MCP-1 upregulation and to determine whether the same trends observed in the RNA expressions exist at the protein level, we selected IL-6 and TNF-α for subsequent ELISA assays. Following the addition of both DZNep and HG treatments, HRECs demonstrated a significant induction of IL-6 compared to HG-treated HRECs (Fig. 4G), suggesting a positive correlation with the RNA expressions observed in Fig. 4C. On the other hand, TNF-α protein levels were significantly decreased in the presence of DZNep + HG, as compared to HG-treated HRECs, which is in parallel to the trends observed with TNF-α RNA levels (Fig. 4H). Our DZNep findings demonstrate that PRC2 activity may have an important role in impacting MALAT1, TNF-α, IL-6, IL-1β, and MCP-1 expressions. However, of note, the effect of PRC2 depletion on the expression of IL-6, IL-1β and MCP-1 may also result from an indirect effect of decreased TNF-α levels, which needs further exploration.

### Transient HG treatment does not alter methylation status of the CpG island in *MALAT1* promoter

**Figure 5:**
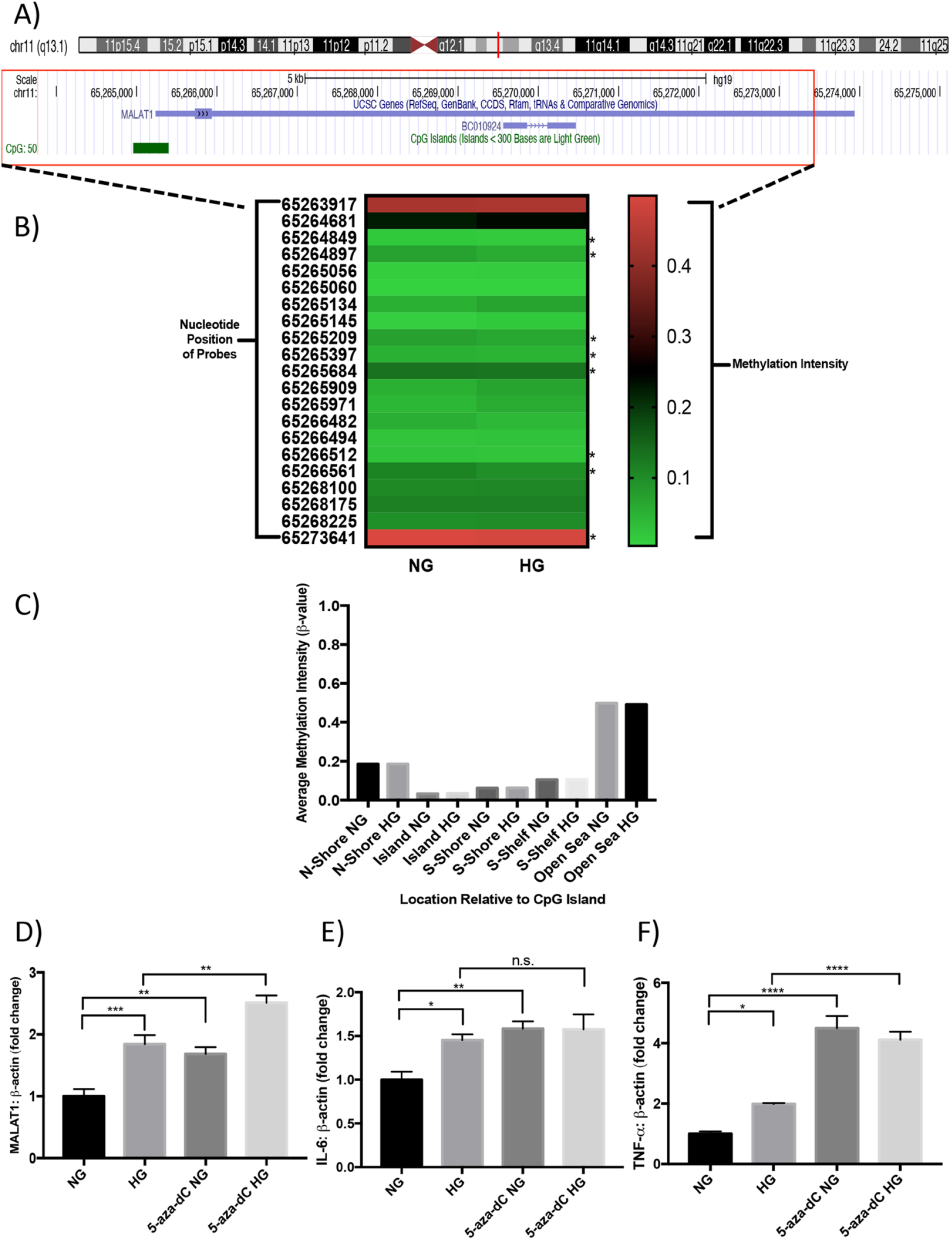
Transient HG treatment does not alter methylation status of CpG island in *MALAT1* promoter; however, DNA methylation inhibition augments glucose-induced upregulations of MALAT1, TNF-α, and IL-6 expressions. (**A**) Information on the human *MALAT1* gene according to the UCSC database. The *MALAT1* gene is 8,707 nucleotides in length (nucleotide positions in chromosome 11.q13.1: 65,265,233 to 65,273,939) and contains a CpG island in its promoter region that is located from positions 65,264,958 to 65,265,398 (441 nucleotides in size)^68^. (**B**) An intensity map depicting the *β*-values of CpG sites across the *MALAT1* gene, generated by the Illumina methylation array. A *β*-value of 0 indicates no methylation, while a value of 1 indicates complete methylation at the interrogated site (the ‘*’ demonstrates a reduction in methylation following HG treatment). (**C**) A bar graph illustrating the *β*-values separated by regions relative to the CpG island in the *MALAT1* promoter. These regions are defined as ‘Island’ (an area of at least 500 base-pairs that contains an observed-to-expected CpG ratio greater than 60%), ‘Shore’ (areas 2 kilo-bases on either side of an island), ‘Shelf’ (areas 2 kilo-bases on either side of a shore), or ‘Open Sea’ (areas outside of the shelf). No significant differences were observed in methylation between NG and HG conditions in these regions (data expressed as average *β*-value per region; *n = *3 independent samples for each NG or HG group). Glucose-induced elevations of (**D**) MALAT1, (**E**) IL-6, and (**F**) TNF-α transcripts were further increased following 5-aza-dC treatment (data expressed as a ratio to β*-actin* (mean ± SEM); normalized to NG; *P < 0.05, **P < 0.01, ***P < 0.001, ****P < 0.0001, and n.s. = not significant, compared to NG or HG; *n* = 6 from three independent experiments and performed in triplicates).

To address whether differential methylation patterns exist in the *MALAT1* promoter in hyperglycemia, we cultured HRECs in NG and HG conditions and performed a genome-wide methylation analysis. Following detection of genome-wide methylation (over 830,000 methylation sites), we filtered to specifically examine the sites that spanned across the *MALAT1* gene, which amounted to 21 probes (Data File S1). We found that the average methylation intensity was generally lower across the ‘Shore’, ‘Island’, and ‘Shelf” regions in both NG and HG-treated HRECs (β-values < 0.2) compared to the ‘Open Sea’ region, **(**β-values = ~0.49, Fig. 5C). Furthermore, in both NG and HG conditions, HRECs demonstrated the lowest degree of methylation in the CpG islands compared to the other regions (β-values < 0.074, Fig. 5C). Although eight probe sites indicated a slight reduction in methylation after HG treatment (Fig. 5B), no comparable differences in methylation were demonstrated overall between HRECs in NG and HG conditions (Fig. 5C).

### Inhibition of DNA methyltransferases (DNMTs) increases expression of MALAT1 and inflammatory cytokines

We then examined the effects of inhibiting genome-wide DNA methylation on MALAT1, TNF-α, IL-6, IL-1β, and MCP-1 RNA expressions. To produce such an environment *in vitro*, we administered the pan-DNMT inhibitors 5-aza-dC or zebularine to HRECs prior to glucose treatment and analyzed the effects of methylation loss on RNA expressions using RT-qPCR. Following these treatments, we confirmed the reductions of DNMT1, DNMT3A, and DNMT3B (Fig. S6A–C and F–H). Interestingly, inhibiting the activity of DNMTs in NG and HG conditions evoked further increases in MALAT1, IL-6, TNF-α, IL-1β, and MCP-1 RNA expressions when compared to control NG and HG-treated HRECs (Figs 5D–F, S6D,E and I–M). Among the markers analyzed, TNF-α and IL-1β demonstrated the greatest upregulations in 5-aza-dC or zebularine-treated HRECs (Figs 5F and S4E). The findings implicate that although transient HG treatment on HRECs may not generate considerable DNA methylation alterations across the *MALAT1* gene region, globally inhibiting the activity of DNMTs impacts the expression of MALAT1 and inflammatory transcripts. Therefore, we speculate that DNMTs may have a potential role in regulating *MALAT1* and inflammatory RNA expressions, which needs further characterization.

To confirm the findings from our pan-DNMT inhibitors, we selected DNMT1, a constitutively expressed DNMT, for subsequent siRNA-mediated knockdown (Fig. S7A). After silencing DNMT1, we observed overall increases in MALAT1, TNF-α, IL-6, IL-1β and MCP-1 RNA expressions in both NG and HG-treated HRECs (Fig. S7B–F).

**Figure 6:**
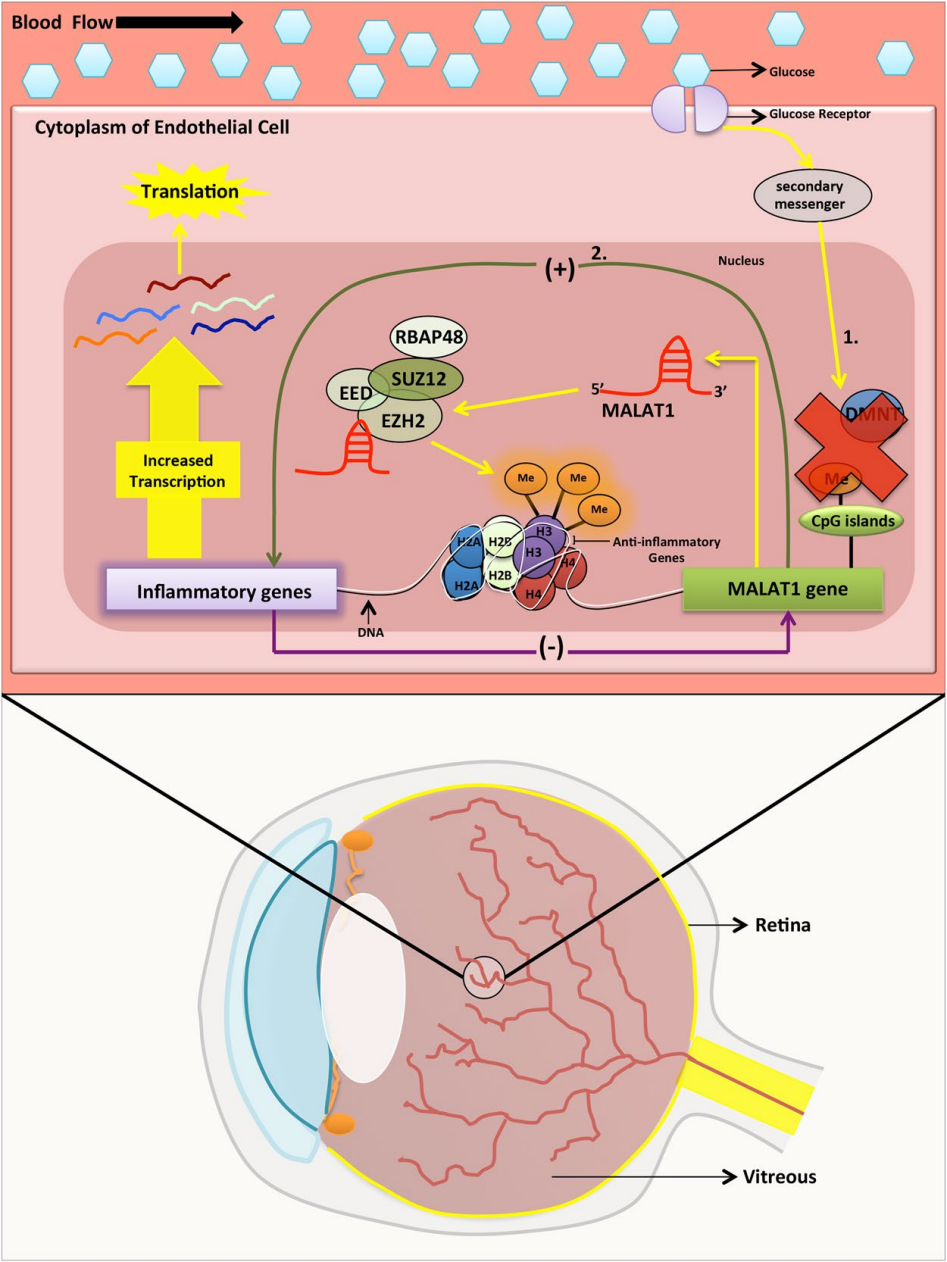
A schematic depicting the potential involvement of MALAT1 in regulating inflammation through epigenetic mechanisms in diabetic retinopathy. Based on our findings, we present a diagram for MALAT1 in potentially regulating inflammation by independent and dependent pathways in DR. In the independent pathways, indicated by ‘1’, DNA methylation regulates the transcriptional status of *MALAT1* and if the actions of DNA methyltransferases are hindered, MALAT1 RNA expressions will increase. Following upregulation, MALAT1 can recruit PRC2 to the promoters of anti-inflammatory genes and epigenetically repress these targets, which will subsequently allow for heightened transcription of inflammatory genes. On the contrary, in the dependent pathway (indicated by ‘2.’), the MALAT1 transcript and *MALAT1* gene may directly interact with inflammatory transcripts and inflammatory genes, respectively, to ultimately provoke a greater inflammatory response (Me = methylation; EZH2 + SUZ12 + EED + RBAP48 = PRC2 complex components; (+) and (−) = feedback loop; 1. = Indirect effect of MALAT1 on inflammation via DNA methylation and PRC2 pathways; and 2. = Direct effect of MALAT1 gene activity on inflammation and vice versa).

Based on our findings, we propose a diagram for MALAT1 in potentially regulating inflammation through independent and dependent pathways in DR (summarized in Fig. 6). In the independent pathways, DNA methylation may be capable of regulating the transcriptional status of *MALAT1* and if the actions of DNMTs are hindered, MALAT1 RNA expressions could increase. Following upregulation, MALAT1 may recruit PRC2 to the promoters of anti-inflammatory genes and epigenetically repress these targets, which might subsequently allow for heightened transcription of inflammatory genes. On the contrary, in the dependent pathway, the MALAT1 transcript and *MALAT1* gene may directly interact with inflammatory transcripts and inflammatory genes to ultimately provoke a greater inflammatory response.

## Discussion

When metabolic memory was first described^3,4^, it became clear that understanding the processes implicated in this phenomenon are of utmost importance. Epigenetic modifications, which can alter the expression of genes without altering the underlying DNA sequence, are critical players in metabolic memory^35^ and characterizing these modifications will help us learn about the intricacies of epigenetic regulation in diseases. Recently, the advancements in experimental genome-wide approaches have allowed for the identification of lncRNAs, which play various roles in cellular physiology and are heavily involved in epigenetic regulation^35,36^. Further insight into the functions of lncRNAs and their association with other epigenetic mechanisms in diseases will help with the development of better-targeted therapeutics. Here, not only do we show for the first time that the lncRNA MALAT1 is present in the VH of diabetic patients, but we present through a series of well-designed *in vitro* and *in vivo* experiments, a novel epigenetic paradigm for MALAT1 in the pathogenesis of DR.

MALAT1 was originally discovered in patients with non-small cell lung carcinoma and shortly after its discovery, MALAT1 has also been reported in various pathologies such as heart disease and diabetes^10–13,25,26,37^. Located on human Chr.11q13.1, the *MALAT1* gene produces a well-conserved non-coding transcript that is ~8000+ nucleotides in length and is further processed by RNAses P and Z, which ultimately results in the production of two transcripts: a mature MALAT1 transcript and a ~61-nucleotide MALAT1-associated small cytoplasmic RNA (mascRNA)^11,22^. The mature MALAT1 transcript is primarily localized to the nucleus, while the mascRNA is exported to the cytoplasm^22–24,38^.

The stability of the mature MALAT1 transcript differs across cell-types, where MALAT has been reported to have a half-life of ~9 hours in human cervical cells (HeLa Tet-off)^39^, 16.5 hours in human B cells^40^, and 3 hours in murine NIH 3T3 cells^40^. Of note, we have previously reported in HUVECs that MALAT1 expression levels increase significantly at 12 hours^12^; whereas, other reports have indicated that MALAT1 expression is highest at the 48-hour mark in RF/6A, primary retinal ganglion, and Müller cells^10,26^. Our current results indicate that MALAT1 expression peaks at the 48-hour mark following HG treatment in HRECs, which is in accordance with the findings from Liu *et al*. and Yao *et al*.^10,26^. It is likely that MALAT1 transcripts may exhibit similar half-life patterns among specific retinal cells, which may explain the variation in MALAT1 expression between HUVECs and HRECs. As well, early increases of MALAT1 by HG may regulate specific pre-mRNA splicing patterns and potentially activate a degradation mechanism that adjusts the MALAT1 expression at later time points^41^. Nevertheless, it is important to note that although the initial increase of MALAT1 at 48 hours may appear as a temporary event, MALAT1 may be capable of activating persistent epigenetic changes involving its downstream targets—despite reductions in MALAT1 expression later in the disease course. This phenomenon is best explained by the concept of metabolic memory, which has been previously demonstrated with epigenetic changes occurring in the NF-κB promoter following transient hyperglycemia^42^.

Knocking down lncRNAs using siRNA-mediated approaches has allowed for critical discussion on the efficiency and specificity of these particular knockdowns. Despite the notion that siRNAs operate mainly in the cytoplasm, several studies have demonstrated that siRNA-programmed RNA-induced silencing complexes (RISCs) exist in the nucleus, where siRNAs can still be used to target nuclear lncRNAs^43–45^. In fact, previous studies have demonstrated efficient MALAT1 knockdown using siRNA-mediated transfections^13–19,25,26,38^. As indicated by our prior work, the selective targeting of MALAT1 via siRNAs resulted in a subsequent reduction of IL-6 and TNF-α mRNA and protein levels in HUVECs^12^, which also resembles similar patterns observed in this study. A reduction in MALAT1 with concomitant decreases in inflammatory transcripts suggests that MALAT1 promotes an inflammatory phenotype in DR. Furthermore, in the context of PRC2, we recently demonstrated that knocking down lncRNA ANRIL downregulates the RNA expressions of PRC2 components in HG-treated HRECs^46^. Since we observed similar trends in PRC2 expressions after siMALAT1 treatment, our findings collectively support the notion that certain lncRNAs, such as ANRIL and MALAT1, may act as scaffolds to chromatin-associated complexes in order to further modulate the expression of genes in DR^47^.

Under normal physiological conditions, the *Malat1* KO mice do not display a noticeable phenotype^22–24^. However, whether *Malat1* KO mice will reveal a disease-specific phenotype, when subjected to certain pathological conditions, remains of great interest. With respect to our *in vivo* diabetic animal model, our initial findings of MALAT1 upregulation in the WT-D mice retinas at 2 months are consistent with previous demonstrations of increased MALAT1 expression in the retinas of diabetic rats^25,26^. However, of note, the relative MALAT1 expressions documented from these studies were upregulated up to >3-fold at the aforementioned time points^25,26^; whereas in our study, the relative MALAT1 RNA expression significantly increased to ~1.46-fold in the WT-D retinas at 2 months. We believe the differences observed in MALAT1 expressions may be accounted for potential species-specific or other unidentified variations^48,49^. Since we also observed statistically significant reductions in the expressions of IL-6, TNF-α, IL-1β, MCP-1, EZH2, SUZ12, and EED in the diabetic *Malat1* KO retinas, we infer that MALAT1 may be functionally capable of controlling the expressions of PRC2 components, through which it may regulate inflammation in DR. To further elaborate on the potential transcriptional capabilities of MALAT1, intergenic lncRNA transcripts have been previously shown to modulate the expression of protein-coding genes in *cis* by reducing the recruitment of transcription factors in the promoter regions of protein-coding genes and subsequently activating histone modification cascades to silence gene transcription- also known as transcriptional interference^50–52^. It is also likely possible that prolonged diabetic conditions can enable MALAT1 to act in *trans* by directly impacting RNA polymerase II activity, which could impact the transcription of nearby anti-inflammatory genes^53^. Moreover, confirming the immunohistochemistry findings from our *in vivo* animal model, previous work by Michalik *et al*. demonstrated that MALAT1 is capable of regulating and enhancing angiogenesis in the neonatal mice retina, suggesting a proangiogenic role for MALAT1 during retinal neovascularization^13^. Although the main focus of our manuscript has been on MALAT1 and its epigenetic role in inflammation, future experiments should elucidate the underlying epigenetic mechanisms implicated in the complex crosstalk between inflammation and angiogenesis—as both of these processes converge and synergistically influence the progression of DR^54^.

The close proximity of the VH to the lens and retina make it an ideal location to administer therapeutic agents to target ocular pathologies^29^. Yao *et al*. have previously indicated that MALAT1 expression levels were significantly downregulated in the aqueous humor of patients with primary open-angle glaucoma^26^. However, in patients with Alzheimer’s Disease or glioma, the cerebrospinal fluid and the tumor itself, respectively, showed MALAT1 upregulation^26^. In contrast, Yan and colleagues have reported increased MALAT1 expression in the fibrovascular membranes and aqueous humors of diabetic patients^10^. Previous studies have also shown that MALAT1 upregulation contributes to epiretinal membrane formation in patients with proliferative vitreoretinopathy^55,56^. In keeping with previous data, intraocular administration of MALAT1 shRNA alleviates vascular leakage and dampens the inflammatory response in the diabetic rat retina through reduced protein expressions of TNF-α, intracellular adhesion molecule-1 (ICAM-1), and vascular endothelial growth factor (VEGF)^25^. To our knowledge, this is the first study to directly identify MALAT1 RNA from the VH of PDR patients. Although much is unknown about the specific mechanisms of lncRNAs in diabetic complications, our novel finding suggests the possibility of therapeutically targeting MALAT1 in PDR patients. Taken together, a prolonged diabetic environment can ultimately allow for increased MALAT1 expression at various locations in the eye, which may associate with increased inflammation and subsequent cellular damage^10,13,25,32^.

Although interactions between lncRNAs and chromatin modification complexes have been studied in cancers^57^, such studies have not been undertaken in the context of DR. MALAT1 has been shown to directly bind to EZH2 in renal cell carcinoma^14^, mantle cell lymphoma^17^, prostate cancer^15^, osteosarcoma^16^, and gastric cancer^58^, suggesting an important regulatory relationship between the two components. Of particular interest, Wang *et al*. revealed that MALAT1 could facilitate the targeting capabilities of EZH2 and further augment histone 3 lysine 27 trimethylation (H3K27me3) levels at the target gene loci of EZH2 in prostate cancer cell lines^15^. These findings allude to the many pathogenetic capabilities of MALAT1. Moreover, our RIP findings demonstrate heightened MALAT1 binding with EZH2 in HG-treated HRECs and we have also previously shown similar binding of EZH2 to ANRIL in HRECs cultured with HG^46^—suggesting a potential overlap in functional mechanism. Therefore, despite observing the expected reductions in MALAT1, TNF-α, EZH2, EED, and SUZ12 expression levels after DZNep treatment, it was surprising to see an increase in IL-6, IL-1β, and MCP-1 expressions in HRECs following DZNep treatment. These data are in keeping with Serresi and colleagues, who indicated previously that Polycomb-mediated repression exists on the IL-6 gene in NSCLC by H3K27me3^59^. Furthermore, a similar observation by Lee *et al*. demonstrated that the depletion of EZH2 or estrogen receptor (ER) in ER-positive breast cancer cell lines (T47D and MCF7) produced a significant upregulation of IL-6 and IL-8 expressions at basal levels^60^. In ER-negative breast cancer, EZH2 acts as a coactivator for RelA and RelB and this in turn activates a positive feedback loop through NF-κB signalling pathways to enhance the expressions of NF-κB targeted genes, such as IL-6^60^. Lee and colleagues concluded that EZH2 is capable of context-specific regulation on the expression of NF-κB target genes^60^. These findings are supportive of our data. However, since DZNep inhibits global histone methylation and is not completely selective^61^, further research is warranted on elucidating the cellular-specific mechanisms of DZNep treatment in diabetic complications.

Although we did not observe significant methylation changes in CpG sites across the *MALAT1* gene after HG treatment, we did notice increased expressions of MALAT1, IL-6, TNF-α, IL-1β, and MCP-1 after 5-aza-dC or zebularine treatments in both basal and HG conditions. Consistent with the findings from our methylation array, Hu *et al*. reported no effects of CpG island methylation status on MALAT1 expression in esophageal squamous cell carcinoma (ESCC) cells^19^. On the other hand, Guo *et al*. analyzed the CpG island methylation patterns in the *MALAT1* promoter of lung cancer cells (A549) and observed a reduction of methylated sites in lung cancer cells^18^. They also demonstrated that a methyl donor could reduce MALAT1 expression in these cells—implying that DNA methylation can regulate MALAT1 expression^18^. Taken together, the diverse findings documented from previous DNA methylation reports, and from our study, imply that disease-and cell-specific responses may exist after inhibiting the activity of DNMTs. Despite our observations from this pharmacological experiment, it still remains to be determined whether the inhibition of genome-wide DNA methylation alters neighbouring (or distal) genes, which in turn may substantially impact the transcriptional activity of *MALAT1*. Future studies should include targeted genome editing techniques such as CRISPR-Cas9, to eliminate any potential off-targeting effects observed from our 5-aza-dC, zebularine, or siDNMT1 experiments.

Overall, our findings collectively demonstrate that MALAT1 is capable of impacting the expressions of inflammatory transcripts through its association with epigenetic mediators, such as histone and DNMTs. It is important to note that the findings described in this manuscript are simply a starting point for future investigations to build and develop a more definitive model for lncRNAs in DR. Establishing such an all-encompassing model, which includes the interplay of miRNAs and additional epigenetic modifications, will enable the development of better-targeted treatment strategies.

## Research Design and Methods

### Cell Culture

We utilized HRECs (Olaf Pharmaceuticals, Worcester, MA, USA) for the *in vitro* experiments. The experimental and culture conditions for HRECs were mentioned in our previous studies^46,62^. Briefly, prior to experimentation, HRECs were plated at a density of 4.3 × 10^5^ cells/mL and used between passages 5 and 6 to reduce variability. Cells were grown to 80–90% confluency post-seeding and subjected to serum starvation for 24 hours, which was then followed by the administration of specific glucose levels (normal glucose [NG], 5 mM; high glucose [HG], 25 mM; and osmotic control [LG], 20 mM L-glucose +5 mM D-glucose) at various time points. These glucose levels are based on a large volume of previous experiments^12,32,46,62–65^. All cell culture reagents were purchased from Sigma (Oakville, Ontario, Canada) unless specified and *in vitro* experiments were performed with six replicates and independently repeated at least three times, unless specified.

### Diabetic Mice Model

The Western University Council for Animal Care Committee approved all animals used in this study and the experiments were performed in accordance with *The Guide for the Care and Use of Laboratory Animals* (NIH Publication 85-23, revised in 1996). *Malat1* knockout (KO) mice, with a C57/BL6 background, were obtained through collaboration with Dr. Spector (Cold Spring Harbor Laboratory, Cold Spring Harbor, New York, USA)^22^ and only male mice were randomly organized into control and diabetic groups. Wild-type (WT) non-diabetic and WT diabetic mice were used as comparators to *Malat1* KO non-diabetic and *Malat1* KO diabetic mice. To generate a type 1 diabetic animal model, we used streptozotocin (STZ). STZ injection methods and monitoring have been previously described^63^. At two months following diabetes induction, animals (*n* = 6/group) were euthanized and the retinal tissues were either snap-frozen for future RNA or protein analyses or were placed in 10% formalin for paraffin embedding.

### Clinical Sample Collection

The Western Research Ethics Board at the University of Western Ontario, London, Ontario, Canada, approved the clinical component of this study. Prior to the procurement of surgical specimens, patients provided informed consent and all of the samples were handled in accordance with the *Declaration of Helsinki*. VH was collected from patients undergoing pars plana vitrectomy by an experienced vitreoretinal surgeon. Specimens were then categorized into two groups: diabetic and non-diabetic. The diabetic group comprised of patients diagnosed with proliferative DR (PDR; *n* = 7; 3 males and 4 females; mean age ± SD = 61 ± 6.76 years); whereas, the non-diabetic group consisted of patients that had no previous history of diabetes mellitus and were diagnosed with idiopathic macular hole or a separate non-diabetic ocular condition (*n* = 6; 4 males and 2 females; mean age ± SD = 75 ± 3.52 years). As previously described^64^, VH specimens were centrifuged (12,000 g, 10 minutes, 4 °C) and the pellet was used for RNA extraction using the TRIzol reagent (Invitrogen, Burlington, ON, Canada) and subsequently, real-time quantitative reverse transcription-PCR (RT-qPCR). In order to avoid contaminating RNA from blood cells, indications of vitreal haemorrhage in the VH specimens were immediately excluded from this study.

### Immunohistochemistry

To identify blood-retinal barrier (BRB) damage in the eye, paraffin-embedded mouse retinal sections were applied for immunohistochemical staining of immunoglobulin G (IgG) using anti-mouse IgG antibody (MP Biomedicals, OH, USA), as previously specified^46,65^. Histological slides were evaluated for positive IgG immunoreactivity (arbitrarily scored 0–3, with 3 representing maximum IgG reaction) in a masked manner by an investigator.

### Enzyme-Linked Immunosorbent Assay (ELISA)

In order to measure the cytokine levels from the cell supernatants, human IL-6 and TNF-α ELISA kits were purchased from ALPCO (Salem, NH, USA) and R&D Systems (Minneapolis, MN, USA), respectively. Concentrations for each cytokine were first quantified using the BCA protein assay kit (Pierce, Rockford, IL, USA) and equal protein concentrations were used for each ELISA (100 μg) according to the manufacturer’s instructions. For the IL-6 chemiluminescence assay, the SpectraMax M5 (Molecular Devices, California, USA) was used to detect luminescence. Whereas, for the TNF-α Quantikine ELISA kit, the optical density for each well was determined at 450 nm and corrected at 568 nm using the Multiskan FC Microplate Photometer (Thermo Fisher Scientific, Massachusetts, USA).

### SiRNA Transfection

HRECs were transfected with either pre-designed siRNAs targeting human MALAT1 (ID numbers: n272231 [si1-MALAT1] and n272233 [si2-MALAT1], Life Technologies), DNMT1 (ID number: s4216 [siDNMT1], Life Technologies) or scrambled siRNA (ID number: AM4635, Life Technologies) using Lipofectamine 2000 (Invitrogen, Burlington, ON, Canada) and Opti-MEM (Life Technologies). A lipofectamine-mediated transfection protocol has been indicated in our earlier studies^12,62^. Briefly, 100 μM of siMALAT1, siDNMT1, or scrambled siRNA was used to transfect the cells for 4 hours and subsequently recovered in full medium overnight. The following morning, cells were serum starved for 21 hours and were then incubated with specific glucose concentrations (5 mM or 25 mM) for 48 hours. MALAT1 and DNMT1 knockdown were confirmed using RT-qPCR. Following verification of knockdown, both siMALAT1s demonstrated similar knockdown activity (~75%) when compared to scrambled controls; therefore, we decided to select si2-MALAT1 (ID: n272233) for our subsequent experiments. Furthermore, when compared to scrambled HG controls, siDNMT1 (ID: s4216) demonstrated a ~72% reduction in DNMT1 RNA expression in HG-treated HRECs.

### 3-Deazaneplanocin A (DZNep), 5-Aza-2′-deoxycytidine (5-aza-dC), and Zebularine

Based on previous literature, 5 μM of DZNep (Cayman Chemical, Ann Arbor, MI), 5-aza-dC (Sigma, St. Louis, USA), or zebularine (Cayman Chemical) pre-treatment was applied to HRECs for 1 hour prior to the addition of D-glucose^46,62,66,67^. DZNep, 5-aza-dC, or zebularine-treated HRECs and their respective controls were collected at 48 hours for further analyses.

### CpG Island Methylation Analysis

Whether a differential methylation pattern exists in a diabetic environment remains entirely elusive, therefore we investigated the CpG island methylation status in the promoter of the *MALAT1* gene in NG or HG-treated HRECs. At the 48-hour mark after glucose treatment, HRECs were collected and 1 μg of genomic DNA was used for bisulfite conversion using the EZ DNA Methylation Kit (Zymo Research, Irvine, California, USA). The bisulfite-converted DNA was then hybridized to the Illumina Infinium MethylationEPIC BeadChip array (Illumina, San Diego, California, USA) following the manufacturer’s protocol. To achieve the readout from the array, we used the HiScan System (Illumina, San Diego, California, USA) and subsequently imported the methylated and unmethylated signal intensity data into R 3.4.0 for analyses. The methylation intensity was normalized using the Illumina normalization method with background correction using the minfi package. Probes with a detection *P*-value > 0.01 were excluded from the downstream analyses. Further, probes known to contain SNPs at the single nucleotide extension, or the CpG interrogation, were removed. The beta value (β-value) was used to represent the methylation intensity for each CpG locus and was calculated from the ratio of unmethylated probe to methylated probe, ranging between 0 (no methylation) and 1 (full methylation). Three independent samples were used per group.

### RNA Immunoprecipitation (RIP)

At the 48-hour mark, cell lysates from NG and HG-treated HRECs were collected for immunoprecipitation using the Magna RIP RNA-Binding Protein Immunoprecipitation Kit (Millipore, Etobicoke, ON, Canada) following the manufacturer’s instruction^46^. Anti-IgG (control) and anti-EZH2 antibodies (Millipore) were used to co-precipitate the RNA-binding proteins of interest. The extracted RNAs were then analyzed by RT-qPCR.

### Western Blotting

In order to evaluate the protein expression of EZH2 after siMALAT1 and HG treatments, western blotting was performed. As previously described^62^, cell lysates were obtained from HRECs after 48 hours of NG or HG culture with scrambled or MALAT1 siRNA. The Bicinconinic acid assay (Thermo Fisher Scientific, IL, USA) was used to determine protein concentration, in which 20 μg of protein was used for western blotting. Primary antibody incubation was performed overnight using monoclonal anti-EZH2 (1:500; Millipore) or for 1 hour using polyclonal anti-β-actin (1:10000; Abcam, Toronto, ON, Canada). While, secondary antibody incubation was conducted using anti-mouse IgG (1:5000; Santa Cruz Biotechnology, Santa Cruz, CA, USA) or anti-rabbit IgG (1:5000; Bio-Rad, Hercules, CA, USA) horseradish peroxidase conjugated secondary antibodies. Antigenic detection was performed using enhanced chemiluminescence following the manufacturer’s instruction (GE Healthcare Life Sciences, QC, Canada).

### RNA Isolation and Quantitative Real-Time Polymerase Chain Reaction (RT-qPCR)

Total RNA was extracted using TRIzol reagent (Invitrogen, Burlington, ON) as described^12,46,62–65^. After isolation, RNA concentrations were quantified using a spectrophotometer (260 nm; Gene Quant, Pharmacia Biotech, USA) and 1 μg of total RNA was reverse transcribed to complementary DNA (cDNA) using a high-capacity cDNA reverse-transcription kit (Applied Biosystems, Burlington, ON, Canada). In order to detect RNA expression, cDNA was amplified in the LightCycler 96 System (Roche Diagnostics, Laval, QC, Canada) using SYBR-green master mix (Clontech, Mountain View, CA, USA) and specific primers for the genes of interest (Sigma; Table S1). We analyzed results using the LightCycler 96 SW 1.1 software (Roche) and we calculated expression levels by the relative standard curve method using β*-actin* as an internal control for sample normalization.

### Cell Viability Assay

As previously described^62^, the cytotoxicity of glucose treatments (5 mM and 25 mM) in HRECs were determined using the WST-1 Cell Viability Assay (Roche). Viability was assessed at various durations of incubation (0, 24, and 48 hours). Absorbances were first measured at 450 nm, with the Multiskan FC Microplate Photometer (Thermo Fisher Scientific), and then corrected using 690 nm as the reference wavelength.

### Statistical Analysis

Data are expressed as mean ± SEM, unless specified. To determine statistical significance, GraphPad Prism 7 was used to perform Student’s *t* tests when comparing 2 conditions or 1-way ANOVA followed by Tukey’s post hoc test for multiple comparisons. While, for the clinical samples, non-parametric statistical measures (Mann-Whitney U test) were applied. As well, two-sided Pearson Correlations were performed to determine a linear association between RNA expressions of MALAT1 and PRC2 components, or EZH2 and inflammatory cytokines in HRECs cultured with HG and siMALAT1. Differences below *P* < 0.05 were considered statistically significant.

### Data availability statement

All data generated or analyzed during this study are included in this published article (and its Supplementary Appendix).

## Electronic supplementary material

Supplementary Appendix

Dataset 1
