# Supplementary material for: MALAT1: An Epigenetic Regulator of Inflammation in Diabetic Retinopathy
Source: Sci Rep. 2018 Apr 25;8:6526. doi: 10.1038/s41598-018-24907-w (PMC5916949; doi:10.1038/s41598-018-24907-w)
Supplement: Supplementary file 1 — Supplementary Appendix [file 41598_2018_24907_MOESM1_ESM.docx]

**Supplementary Appendix**

MALAT1: An Epigenetic Regulator of Inflammation in Diabetic Retinopathy

**Saumik Biswas^1^, Anu Alice Thomas^1^, Shali Chen^1^, Erfan Aref-Eshghi^1^, Biao Feng^1^, John Gonder^2^, Bekim Sadikovic^1^, Subrata Chakrabarti^1^***

^1^Department of Pathology and Laboratory Medicine, Western University, London, Ontario, Canada

^2^Department of Ophthalmology, Western University, London, Ontario, Canada

* Corresponding author

Address Correspondence to:

Subrata Chakrabarti
Department of Pathology and Laboratory Medicine

Western University
London, Ontario, N6A 5C1, Canada
DSB 4033
Tel: (519) 685-8500 X36350
Fax: (519) 663-2930

E-mail: subrata.chakrabarti@lhsc.on.ca (SC)

Number of Supplementary Figures: 8

Number of Supplementary Tables: 2

Number of Supplementary Data Files: 1

**Figure S1: MALAT1 and inflammatory transcripts are upregulated in HRECs following high glucose treatment.** RT-qPCR analyses of A) MALAT1, B) IL-6, C) TNF-α, D) MCP-1 and E) IL-1β expressions in HRECs exposed to 25mM (HG) or 5mM (NG) glucose over a time course of 72 hours. MALAT1, IL-6, TNF-α, MCP-1 and IL-1β are upregulated in HG-treated HRECs at 48 hours compared to NG (*=*P*<0.05, **=*P*<0.01, ***=*P*<0.001, and ****=*P*<0.0001 compared to NG; data expressed as mean ± SEM; *n*=6/group; normalized to β-actin, and data represented as a fold change of NG).

**Figure S2: MALAT1 influences MCP-1, IL-1β, and EZH2 expressions and strongly binds with EZH2 in HG-treated HRECs.** RT-qPCR analyses indicating reduced RNA expressions of A) IL-1β and B) MCP-1 following siMALAT1 treatment in HG-treated HRECs (data expressed as a ratio to β*-actin* (mean ± SEM); normalized to SCR NG; *=*P*<0.05, **=*P*<0.01, ***=*P*<0.001, and ****=*P*<0.0001, compared to SCR NG or SCR HG; *n*= 6 from three independent experiments and performed in triplicates). C) Representative Western blot image showing EZH2 expressions, with β-actin as a control, in NG or HG-treated HRECs transfected with scrambled controls or siMALAT1. Western blots presented (in C) were cropped from the same membrane and were cropped to improve clarity. The bands within the range of the molecular marker were retained in this figure and the full-length blots are presented in **Supplementary Figure S5**. D) RNA immunoprecipitation, using an IgG control or EZH2 antibody, demonstrating elevated MALAT1 binding to EZH2 following HG treatment in HRECs. MALAT1 expression was determined by RT-qPCR (*****P*<0.0001 and n.s.= not significant compared to IgG controls; data expressed as mean ± SEM; *n*=3/group, and results are from one experiment that is representative of three independent experiments). SCR= scrambled siRNA; NG= 5 mM D-glucose; HG= 25 mM D-glucose; SiM1= siMALAT1 treatment.

**Figure S3: Positive correlations between PRC2 components and MALAT1 expression, and between EZH2 and inflammatory transcripts after MALAT1 knockdown in HG-treated HRECs.** Pearson correlations between RNA expressions of A-C) MALAT1 and the PRC2 components, and between D-E) EZH2 and the inflammatory cytokines in HRECs following siMALAT1+HG treatments. RNA expressions were normalized to β-actin and *n*=6 in the HG+siMALAT group.

**Figure S4: IL-1β and MCP-1 transcript expressions are impacted by *Malat1* knockout and histone methyltransferases.** RT-qPCR analyses of the retinas from animals, following two months of poorly controlled diabetes showed increased expressions of A) IL-1β and B) MCP-1 inflammatory transcripts in WT-D retinas compared to WT-C retinas. *Malat1* KO prevented such increases in the M1 KO-D group (data expressed as a ratio to β*-actin* (mean ± SEM); normalized to WT-C; *=*P*<0.05, **=*P*<0.01, ***=*P*<0.001, and ****=*P*<0.0001, compared to WT-C or WT-D; *n*= 6/group). Following DZNep and HG treatments, RT-qPCR findings indicate elevations of C) IL-1β and D) MCP-1 RNA expressions compared to NG at 48 hours (data expressed as a ratio to β*-actin* (mean ± SEM); normalized to NG; *=*P*<0.05 and **=*P*<0.01 compared to NG or HG; *n*= 6 from three independent experiments and performed in triplicates). WT-C= Wild-type control; WT-D= Wild-type diabetic; M1 KO-C= *Malat1* KO control; and M1 KO-D= *Malat1* KO diabetic

**Figure S5: Full length blots of Figure S2C.** Red dotted lines indicating the cropping locations. A) β-actin was detected first prior to measuring EZH2 expressions, which were detected the following day. B) Blot reveals EZH2 expressions, as well as residual β-actin expressions from the first imaging run.

**Figure S6:** **Global inhibition of DNMTs significantly elevates the RNA expressions of MALAT1, TNF-α, IL-6, MCP-1 and IL-1β.** HRECs pre-treated with 5-aza-dC demonstrated overall reductions in A) DNMT1, B) DNMT3A, and C) DNMT3B transcripts. While, glucose-induced elevations of D) IL-1β and E) MCP-1 transcripts were further increased following 5-aza-dC treatment. HRECs were also incubated with zebularine (another pan-DNMT inhibitor) and demonstrated similar trends. Reductions in F) DNMT1, G) DNMT3A, and H) DNMT3B transcripts were observed; whereas, I) MALAT1, J) TNF-α, and K) IL-6, L) IL-1β, and M) MCP-1 RNA expressions increased following zebularine treatment (*=*P*<0.05, **=*P*<0.01, ***=*P*<0.001, ****=*P*<0.0001, and n.s.= not significant compared to NG or HG controls; data expressed as mean ± SEM; *n*=6/group; normalized to β-actin, and data represented as a fold change of NG). ZEB= Zebularine.

**Figure S7:** **DNMT1 knockdown impacts MALAT1, TNF-α, IL-6, MCP-1 and IL-1β transcripts.** HRECs transfected with siDNMT1 demonstrated significantly decreased A) DNMT1 transcript expression and increased RNA expressions of B) MALAT1, C) TNF-α, D) IL-6, E) IL-1β, and F) MCP-1 compared to scrambled controls (*=*P*<0.05, **=*P*<0.01, ***=*P*<0.001, ****=*P*<0.0001, and n.s.= not significant compared to SCR controls; data expressed as mean ± SEM; *n*=6/group; normalized to β-actin, and data represented as a fold change of NG). HRECs were also incubated with 5 μM Zebularine (a DNA methylation inhibitor) and demonstrated similar increases in D) MALAT1, E) TNF-α, and F) IL-6 RNA compared to controls (*=*P*<0.05, **=*P*<0.01, ***=*P*<0.001, and ****=*P*<0.0001 compared to SCR NG or SCR HG; data expressed as mean ± SEM; *n*=6/group; normalized to β-actin, and data represented as a fold change of NG). SiDNMT1= siDNMT1 treatment.

**Figure S8: Cell viability of retinal endothelial cells following glucose treatments across various durations of incubation.** WST assay showing no significant differences in human retinal endothelial cell viability between the two groups following various durations of incubation (data presented as mean± SD; n.s.= no significance compared to NG; and n=6/group). Legend: NG= normal glucose, HG= high glucose.

**Figure S1**
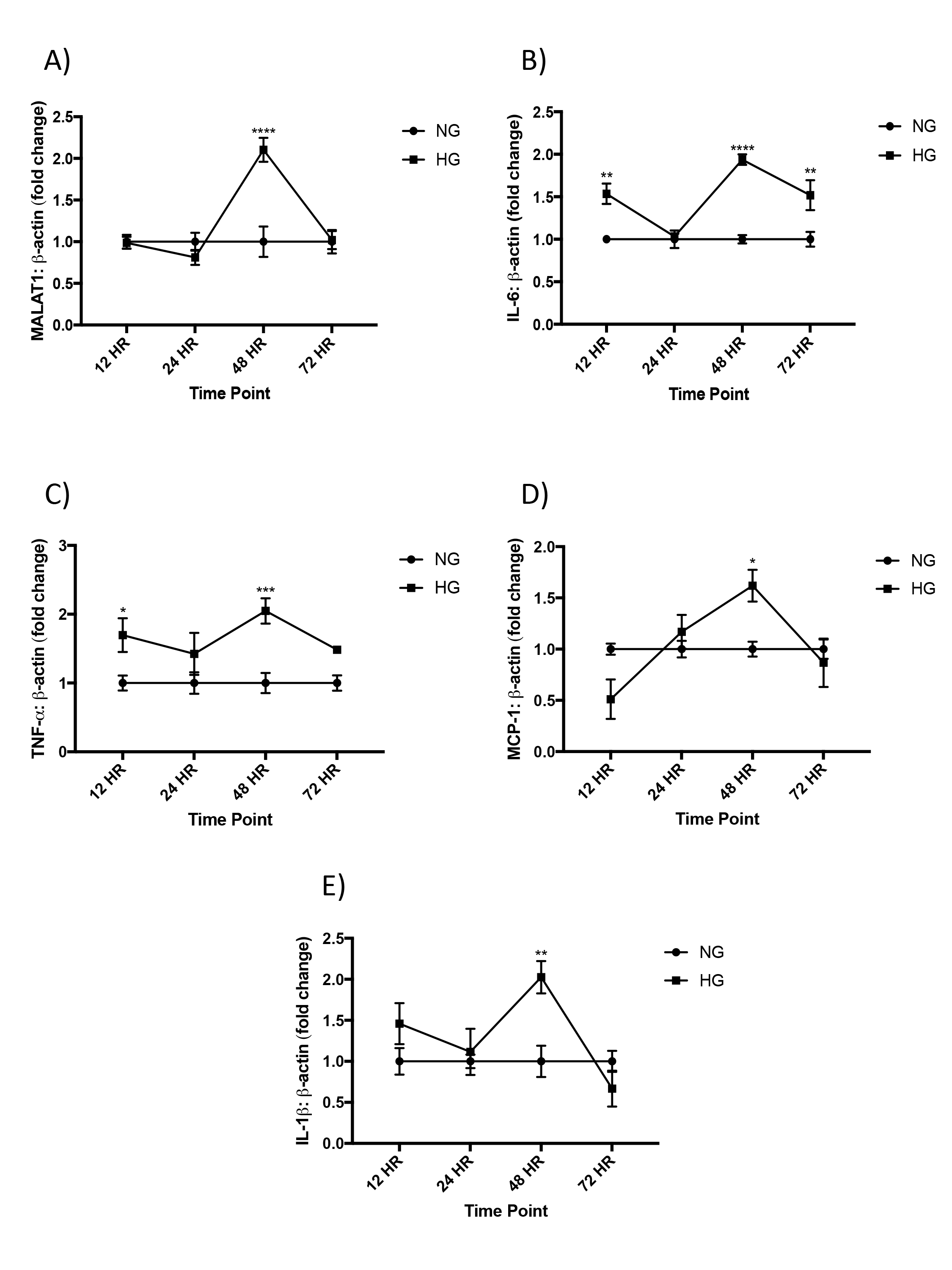


**Figure S2

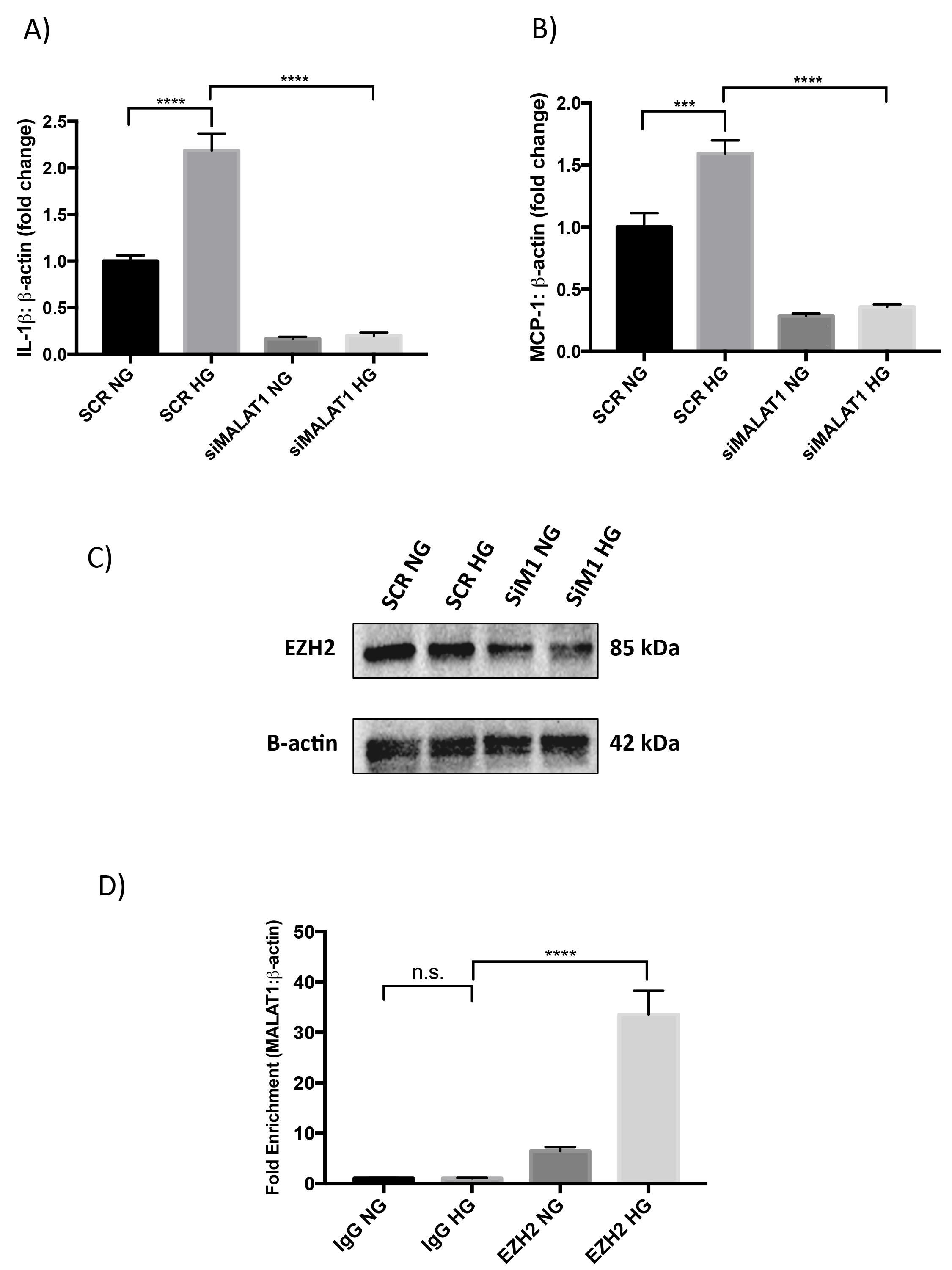
**

**Figure S3

 MALAT1 + HG-treated HRECs

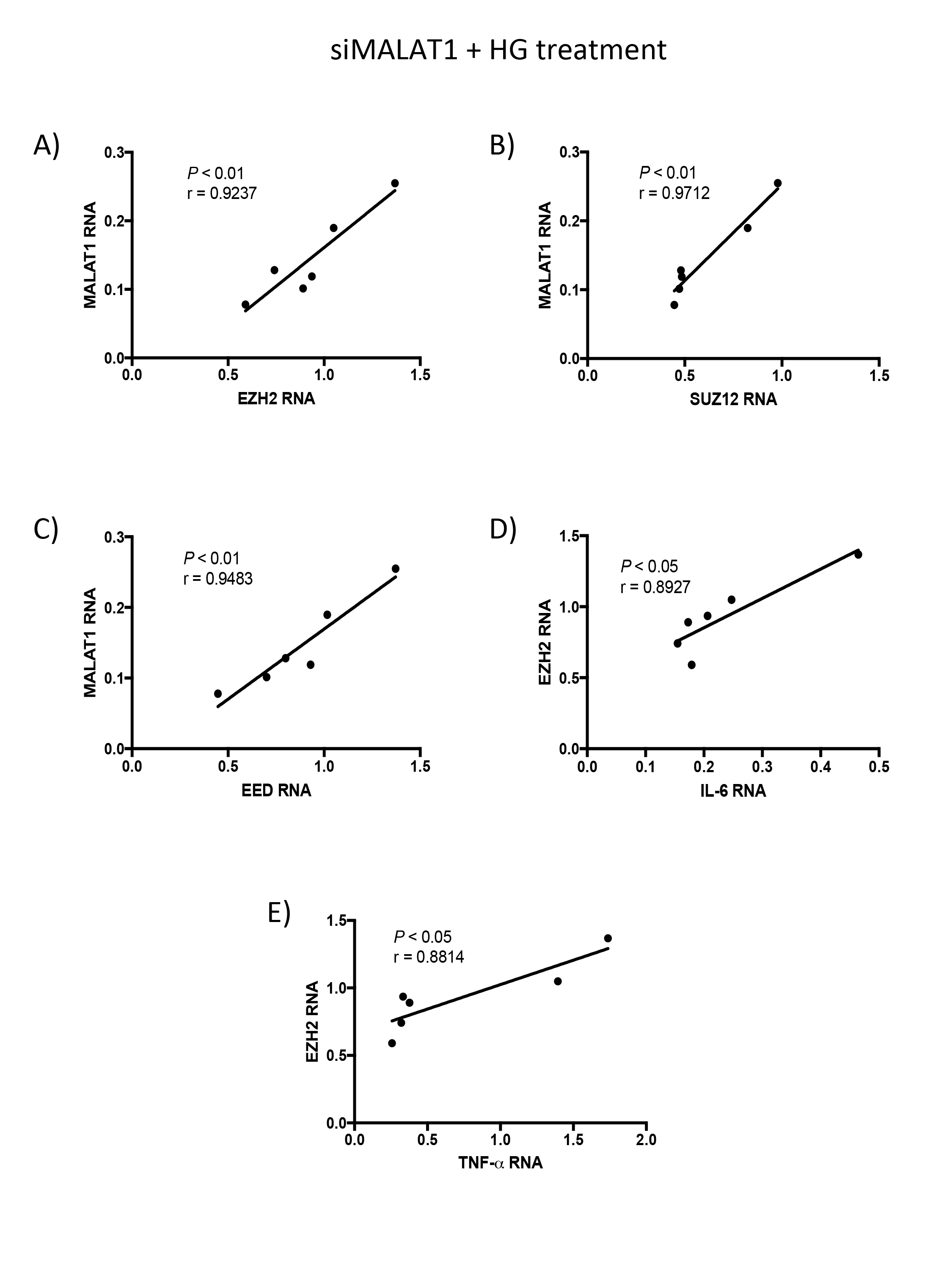
**

**Figure S4:**

**
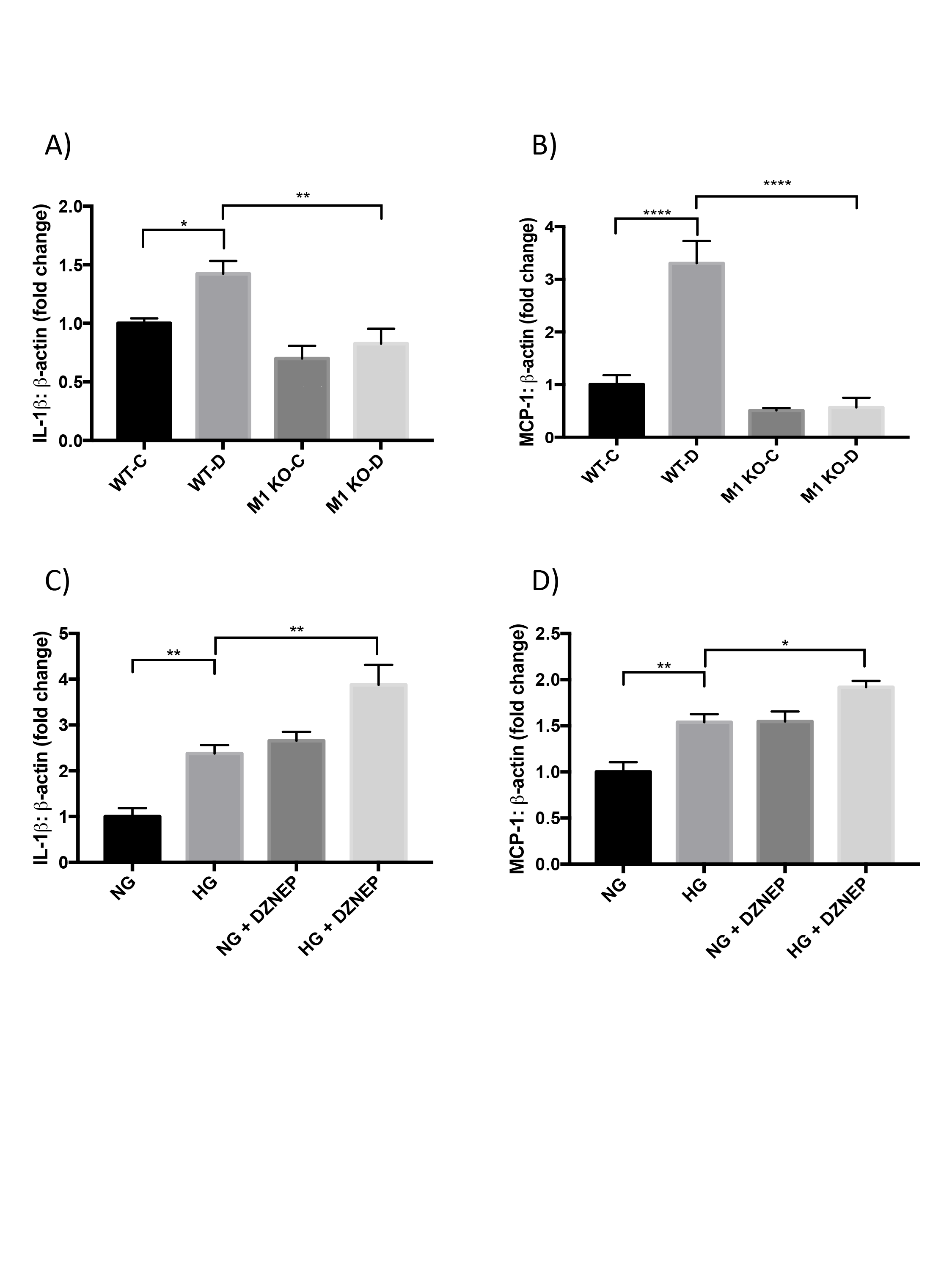
**

**Figure S5:**

**
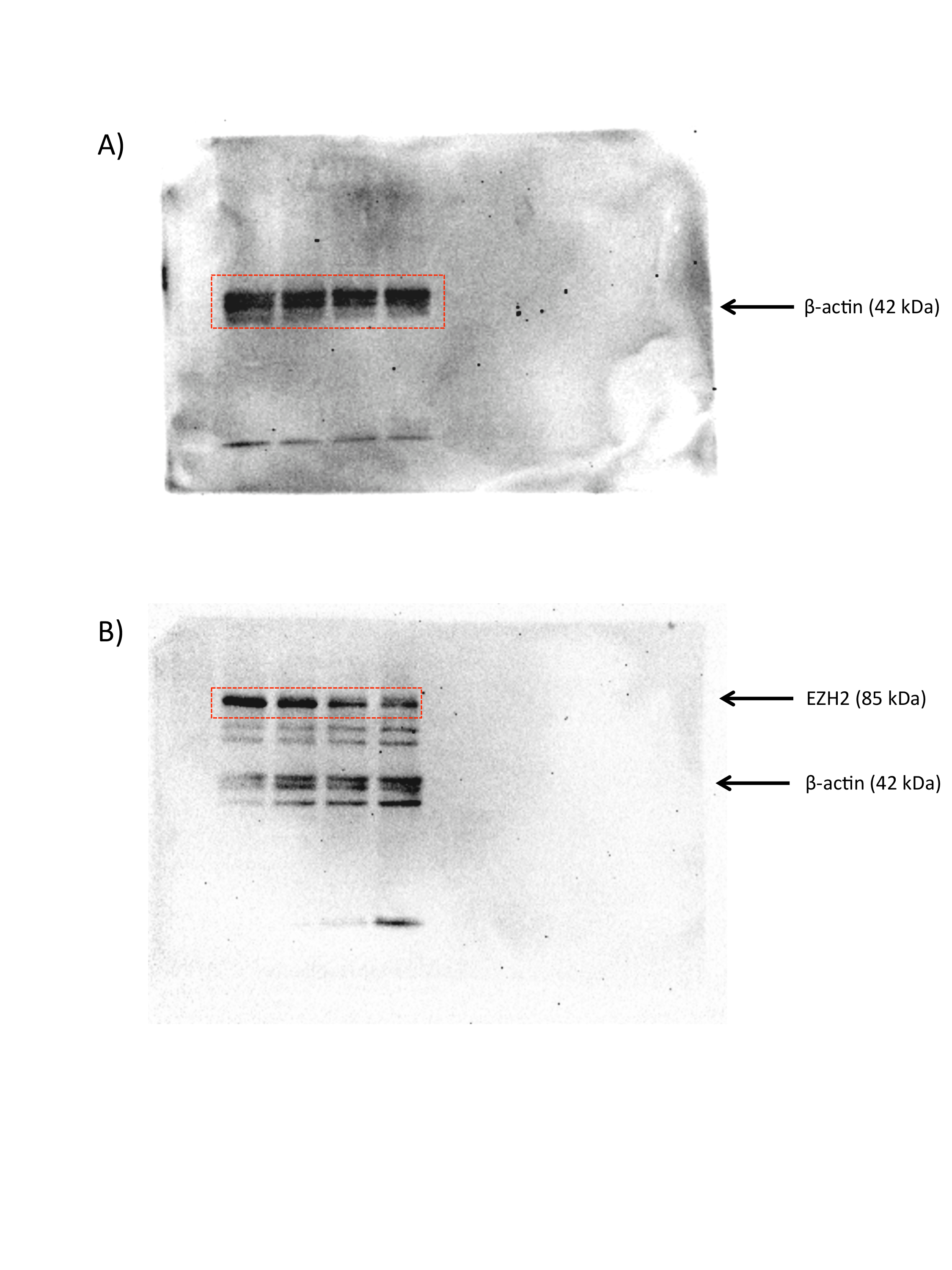
**

**Figure S6:**

**
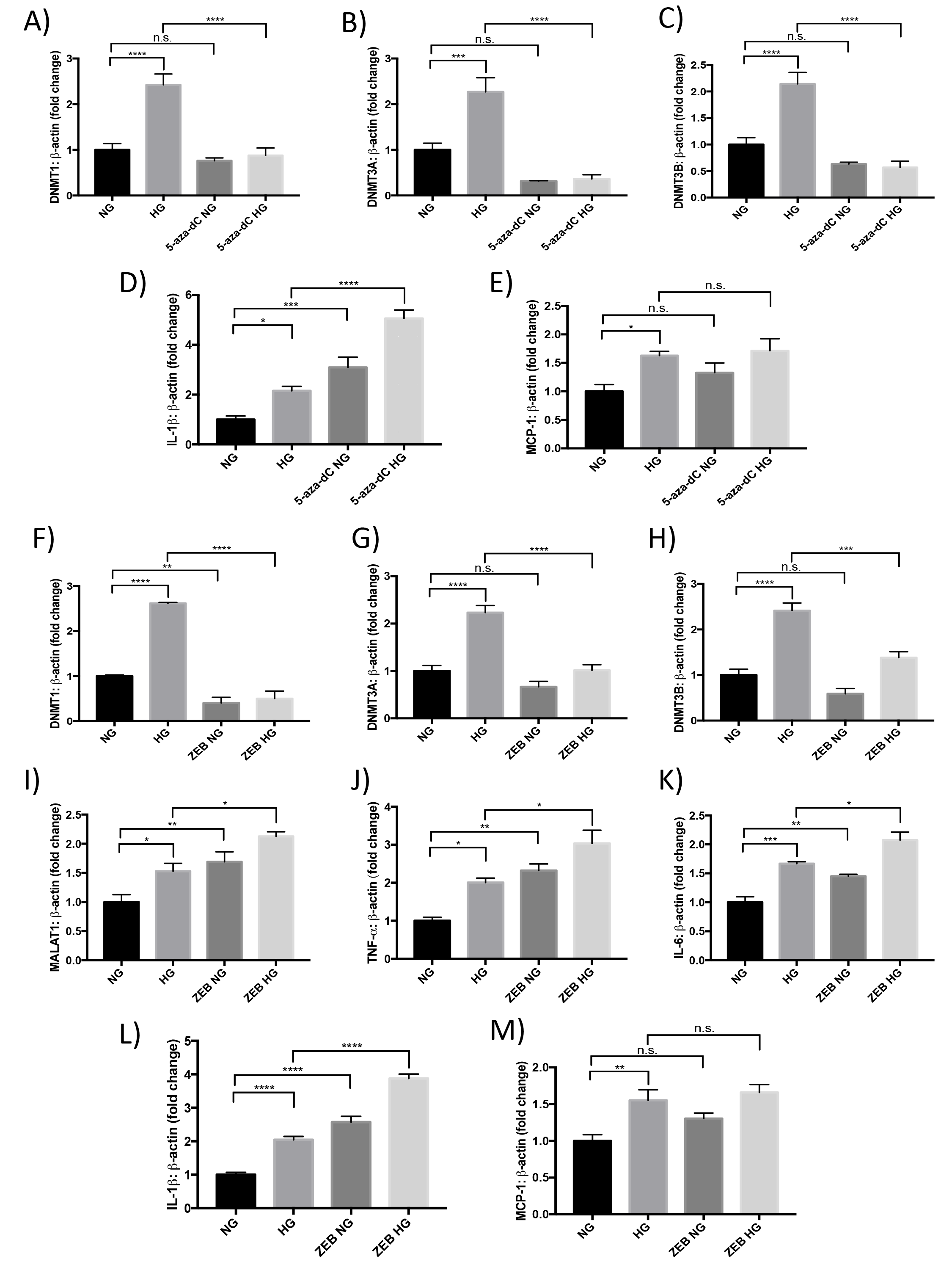
**

**Figure S7:

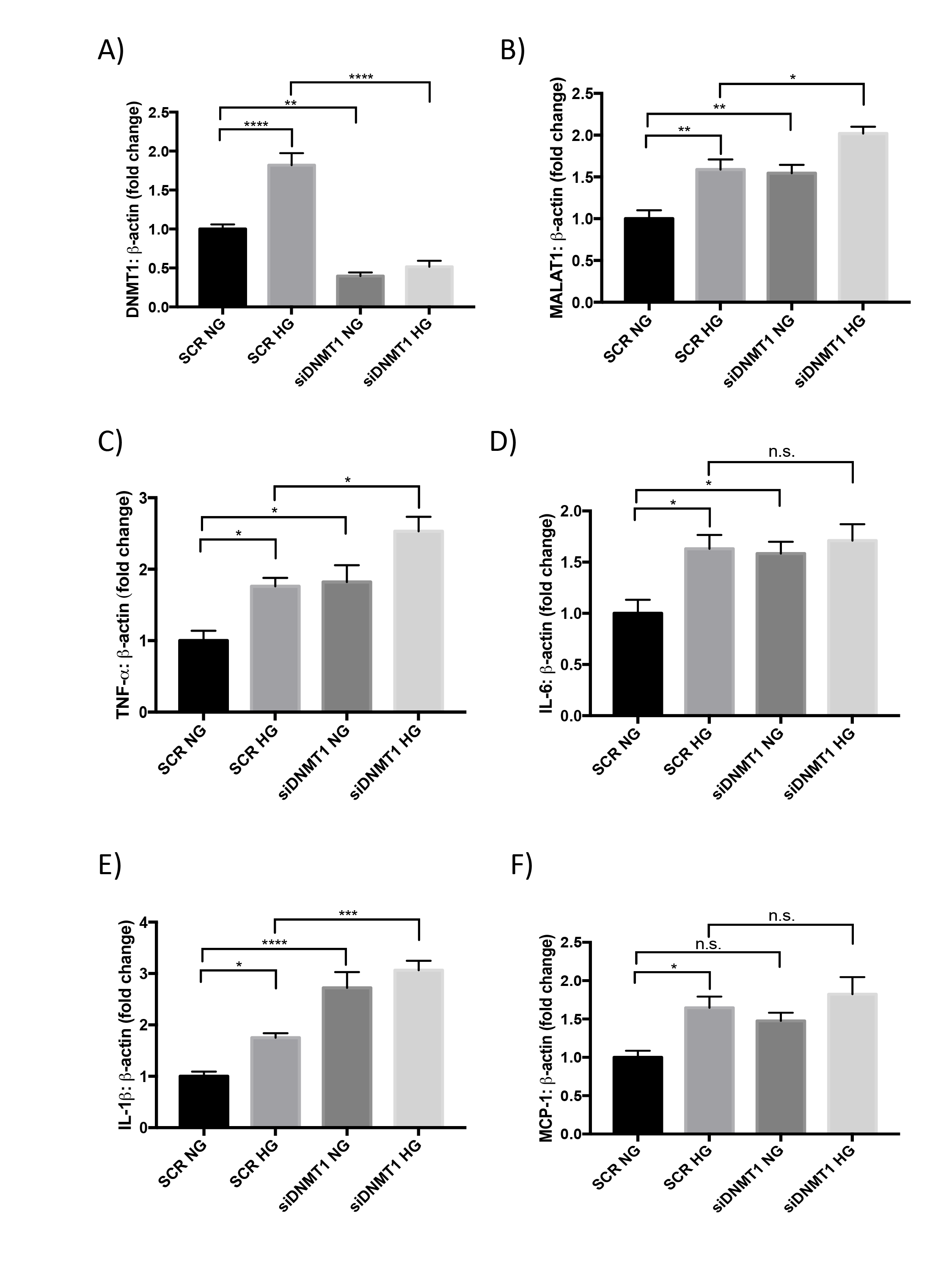
**

**Figure S8:**

**Table S1: Specific oligonucleotide sequences used for RT-qPCR.

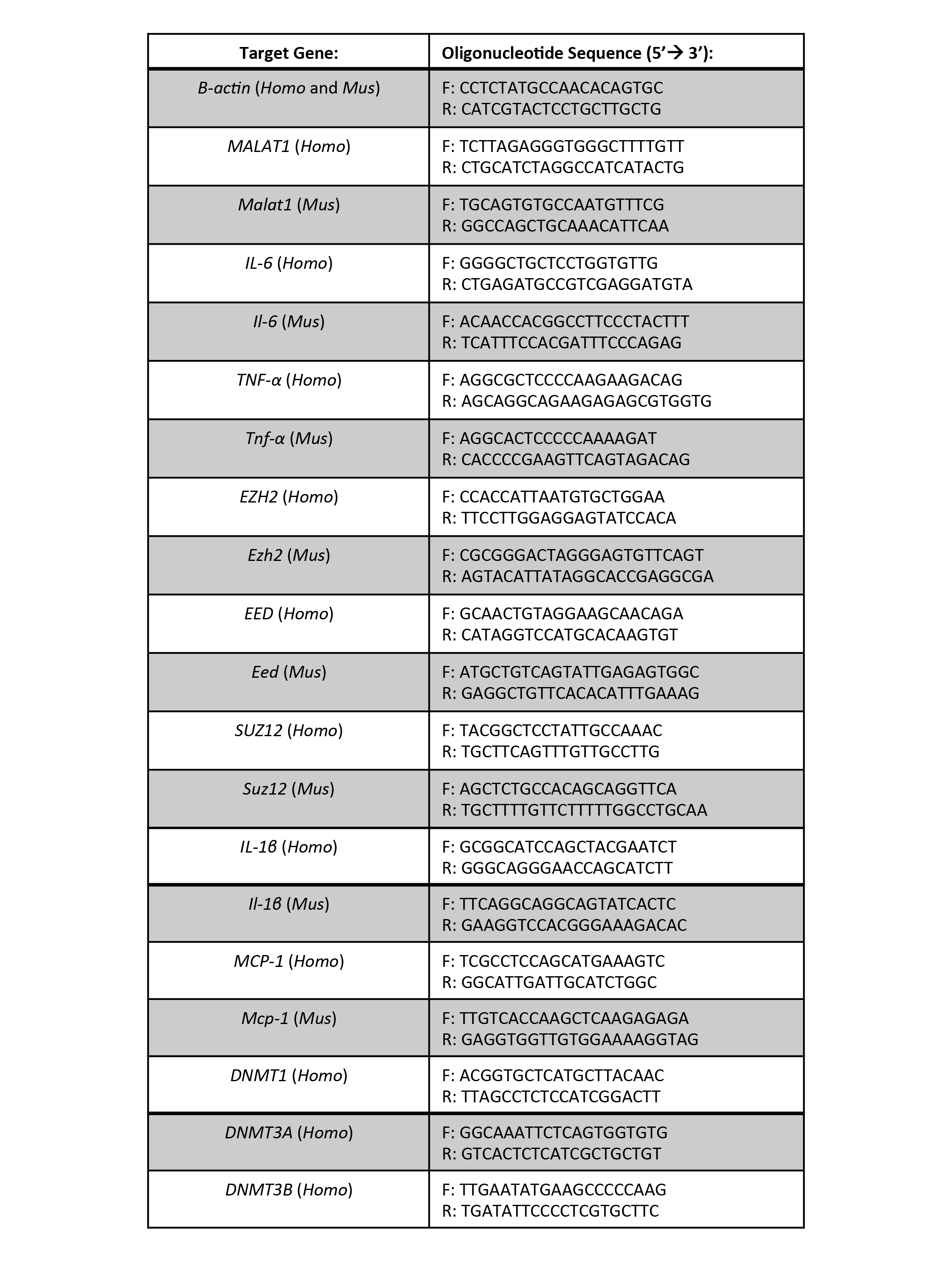

Table S2: Body weights and blood glucose levels for the mice at 2 months.** Legend: WT-control=WT-C, WT-diabetic= WT-D, *Malat1* KO-control= *Malat1* KO-C, and *Malat1* KO-diabetic= *Malat1* KO-D [data expressed as mean ± SEM; *n*=6/group; and ‘*’=significantly different from WT-C].

**
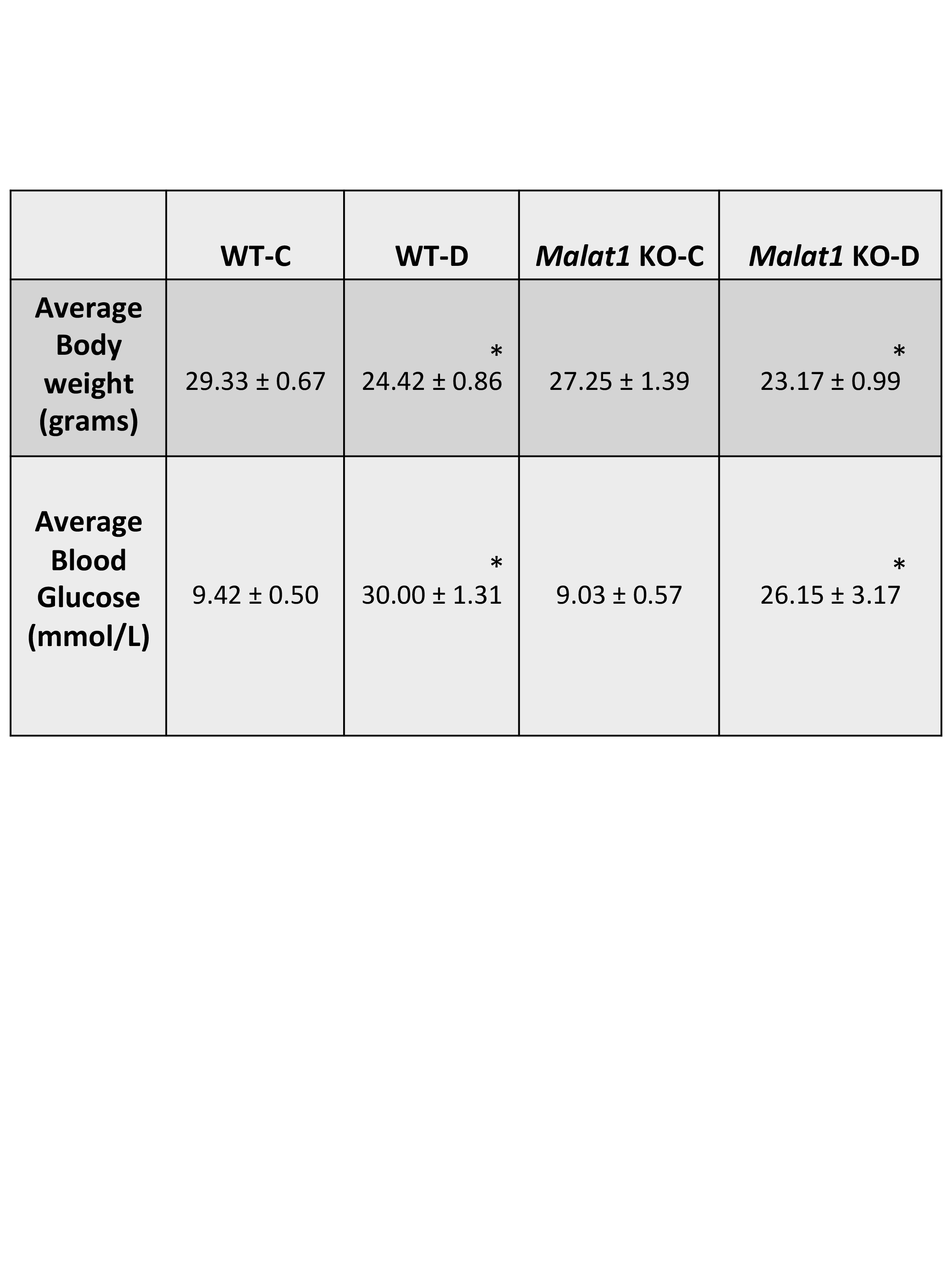
**

**Supplementary Data File 1: Excel file of methylation probes for CpG sites across *MALAT1*.**
